# Dental Calculus Formation Is Linked to Diet and Phylogeny in Mammals

**DOI:** 10.1002/ece3.74105

**Published:** 2026-07-24

**Authors:** John L. Richards, Markella Moraitou, Ella Nates, Konstantina Saliari, Emmanuel Gilissen, Zena Timmons, Andrew C. Kitchener, Olivier S. G. Pauwels, Richard Sabin, Phaedra Kokkini, Roberto Portela Miguez, Katerina Guschanski

**Affiliations:** ^1^ Institute of Ecology and Evolution, School of Biological Sciences University of Edinburgh Edinburgh UK; ^2^ Natural History Museum Vienna Vienna Austria; ^3^ Department of Evolutionary Biology University of Vienna Vienna Austria; ^4^ Royal Museum for Central Africa Tervuren Belgium; ^5^ Alzheimer and Other Tauopathies Research Group, ULB Center for Diabetes Research (UCDR) Université Libre de Bruxelles Brussels Belgium; ^6^ Department of Natural Sciences National Museums Scotland Edinburgh UK; ^7^ School of Geosciences University of Edinburgh Edinburgh UK; ^8^ Royal Belgian Institute of Natural Sciences Brussels Belgium; ^9^ Natural History Museum London London UK; ^10^ Department of Ecology and Genetics/Animal Ecology Uppsala University Uppsala Sweden

**Keywords:** dental calculus, diet, mammals, museum collections, oral microbiome

## Abstract

The oral microbiome is implicated in a wide diversity of fundamental biological functions, with the oral cavity serving as a connection between the host and the external environment. Dental calculus, a mineralized form of dental plaque, preserves the diversity of biomolecules found in the oral cavity through time, serving as a rich source of historical microbiota. Despite its potential, dental calculus has rarely been explored outside of humans and non‐human primates. Hence, it remains unclear how ubiquitous it is across mammals. Using natural history museum collections, we surveyed > 1600 specimens belonging to 142 species, representative of almost all mammalian orders, to investigate the taxonomic distribution of dental calculus, and to identify factors that most strongly contribute to its formation. We found dental calculus to be abundant across mammalian taxa, with 104 surveyed species showing calculus. High‐fiber diets were most strongly associated with calculus abundance, whereas species with high protein and fat diets showed little to no calculus deposits. We found evidence of phylogenetic signal in calculus formation, pointing to the effects of oral/dental morphology. In addition, captivity strongly affected dental calculus formation in almost all dietary categories. Using this information, we made predictions about the likelihood of finding dental calculus in unsurveyed mammalian species, opening doors for its utilization for the study of oral microbiota, past and present. Our study found that dental calculus is well‐preserved and readily available in natural history museum collections, making it an easily accessible source of oral microbiota from wild animals. We highlight the taxonomic diversity of species presenting dental calculus and provide information and suggestions for its use to researchers and curators alike.

## Graphical abstract

In a survey of > 1600 specimens across all mammalian orders, we found that most species present dental calculus. We found calculus production to be strongly associated with herbivory, and we provide recommendations for the use of dental calculus in collections‐based research.

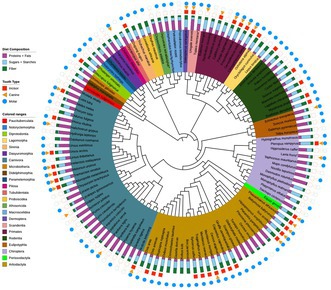

## 1 Introduction

### 1.1 Dental Calculus and Historical Microbiota

Dental calculus (DC) is mineralized dental plaque biofilm, which is visible as calcified deposits on teeth, and provides a valuable source of microbial biomolecules preserved through time (Warinner et al. 2015). Dental calculus is formed through regular, sequential deposition and calcification of the microbial biofilm, trapping other molecules present in the oral cavity, digestive and respiratory tracts, and the environment, reflecting its unique position as a contact point between host internal systems and the outside. Entrapped molecules include dietary phytoliths (Armitage 1975; Power, Salazar‐García, Straus, et al. 2015; Power, Salazar‐García, Wittig, et al. 2015), bacterial, viral, and fungal DNA of the host oral, digestive, and respiratory microbiomes (Brealey et al. 2020; Fellows Yates et al. 2021; Mann et al. 2018), host and dietary DNA and proteins (Hendy et al. 2018; Mackie et al. 2017; Putrino et al. 2024), as well as bioactive inorganic compounds such as heavy metals (Yaprak et al. 2017). This combination of materials makes dental calculus ideal for studying the interplay of host‐associated microbial, dietary, and environmental factors.

The long‐term preservation of diverse (bio)molecules in dental calculus permits investigation across wide temporal scales. Analysis of microscopic remains in dental calculus enabled reconstruction of past diets, informing archaeological research in humans, non‐human primates, and ungulates (Armitage 1975; Ciochon et al. 1990; Hendy et al. 2018). Applications of ancient DNA (aDNA) techniques to historical and ancient metagenomic samples, including preserved microbiomes, have enabled comprehensive studies into the microbiota of the past and their coevolution with their hosts (Brealey et al. 2021; De La Fuente et al. 2013; Fellows Yates et al. 2021; Moraitou et al. 2022; Rivera‐Perez et al. 2015). Dental calculus and paleofeces are the two forms of host‐associated microbiomes that can be preserved through time. Dental calculus has many advantages over paleofeces, as it is far more common, less affected by contamination and decomposition, and can more readily be associated with a host individual (Adler et al. 2013; Warinner et al. 2014). Further, in contrast to paleofeces, the preserved oral microbiome of dental calculus reflects an extended period in the life of the host, likely from months to years, and serves as a representation of historical environments and their effects on host microbiota, as the oral cavity serves as a point of entry for environmental microbes (Brealey et al. 2021; Fellows Yates et al. 2021; Moraitou et al. 2022; Shaiber et al. 2020).

### 1.2 Dental Calculus Formation

The diversity of information captured and preserved through time in dental calculus makes it ideal for addressing a large range of research questions in ecology and evolution and to investigate environmental changes affecting the host species. Despite this great potential, little is known about which species produce dental calculus, and there is a lack of basic knowledge of the factors of host ecology and biology that contribute to its formation. Dental calculus has been studied in several mammal species outside of humans (Armitage 1975; Brealey et al. 2020, 2021; Moraitou et al. 2022; Ozga and Ottoni 2023; Power, Salazar‐García, Straus, et al. 2015; Power, Salazar‐García, Wittig, et al. 2015), but has primarily been considered as a health concern, with the literature on its formation and development being dominated by veterinary studies in pets and livestock (Clarke and Cameron 1998; Hernández‐Castañeda et al. 2015; Verstraete 2003).

In the development of DC, the formation of a biofilm in the oral cavity is first dependent on the colonization of the tooth surface by microorganisms sustained by nutrients present in the oral fluid (Lieverse 1999). The presence of salivary amylase is thought to be important in the development of this initial biofilm layer, as it breaks down starches into simple sugars that form a film adhering to the surface of the tooth. Further fermentation of salivary sugars into acids breaks down the tooth enamel, providing more surface area for biofilm adhesion (Radini et al. 2017). Mineralization occurs through the deposition of calcium phosphate crystals in the biofilm cellular matrix, and the crystal structures strengthen and mature through time, serving, once mineralized, as a surface onto which a new biofilm layer can subsequently adhere and eventually mineralize (Akcalı and Lang 2018).

A range of factors have been proposed to contribute to dental calculus formation, including nutrients, pH, water content, and salivary flow (Jin and Yip 2002; Lieverse 1999). Conflicting reports have attributed heavy calculus formation to high fat, high protein, and high carbohydrate diets, while also highlighting the effects of salivary calcium, phosphate, and pH (see Lieverse 1999). Other factors have the potential to impact dental calculus formation, including tooth morphology, shape of the oral cavity, host behavior, and location of salivary glands (Radini et al. 2017). Many aspects of morphology and feeding behavior that may impact dental calculus formation are strongly coupled with diet but also with host taxonomy. For example, similarities in tooth shape may reflect similarities in diet but also be constrained by evolutionary relationships between hosts (Evans and Pineda‐Munoz 2018).

### 1.3 Dental Calculus in Natural History Collections

Natural history museum collections are ideally suited for the study of dental calculus, as skulls are among the most frequently collected specimens. Dental calculus deposits develop on the surface of the tooth and hence their sampling does not damage the underlying tooth morphology. However, like any destructive sampling, once removed and processed, calculus cannot be recovered. Therefore, both researchers and curators would benefit from a better understanding of which species are likely to reliably form dental calculus so that specific research questions, efficient sampling strategies, and informed future collection foci and specimen processing and storage can be developed for the use of this precious material.

To support researchers in planning their studies and museum curators in accommodating and anticipating sampling requests, we aimed to (i) assess which mammal species develop dental calculus, and to what extent and (ii) uncover underlying principles of dental calculus formation in mammals by considering ecological and phylogenetic aspects of mammalian biology. Lastly, using collected data, we make predictions about dental calculus presence based on host taxonomy and dietary composition, looking towards future developments in the use of dental calculus to study historical microbiota. The potential of dental calculus in reconstructing (past) oral microbiota is immense. In exposing the huge range of as‐yet unexplored sources of dental calculus in a large diversity of species, we hope to highlight the taxonomic and ecological diversity of mammalian host taxa that may be studied.

## 2 Methods

### 2.1 Dataset and Calculus Survey

To assess the prevalence of dental calculus formation across mammals, we surveyed at least one species in each mammalian order for a total of 1629 specimens belonging to 142 species in 129 genera across 72 families (Table S1). Three orders were excluded: Pholidota and Monotremata, as they do not have teeth, and Cingulata, which have a unique, highly reduced tooth morphology, with only a single genus showing reduced enamel formation in some species (Ciancio et al. 2021). We chose to exclude this order due to its unique dental morphology, which differs strongly from that of the other species we surveyed. Surveyed specimens were housed at the National Museums Scotland, Edinburgh, UK (NMS); the Natural History Museum London, UK (NHMUK); the Naturhistorisches Museum Wien, Austria (NHM Vienna); the Royal Belgian Institute of Natural Sciences, Brussels (RBINS); and the Royal Museum for Central Africa, Tervuren, Belgium (RMCA). Species names were recorded as they appeared on specimen labels (Table S1: “Recorded Species”), and specimen taxonomic designations were updated to current accepted classification and verified by museum curators (Table S1: “Accepted Species”).

**FIGURE 1:**
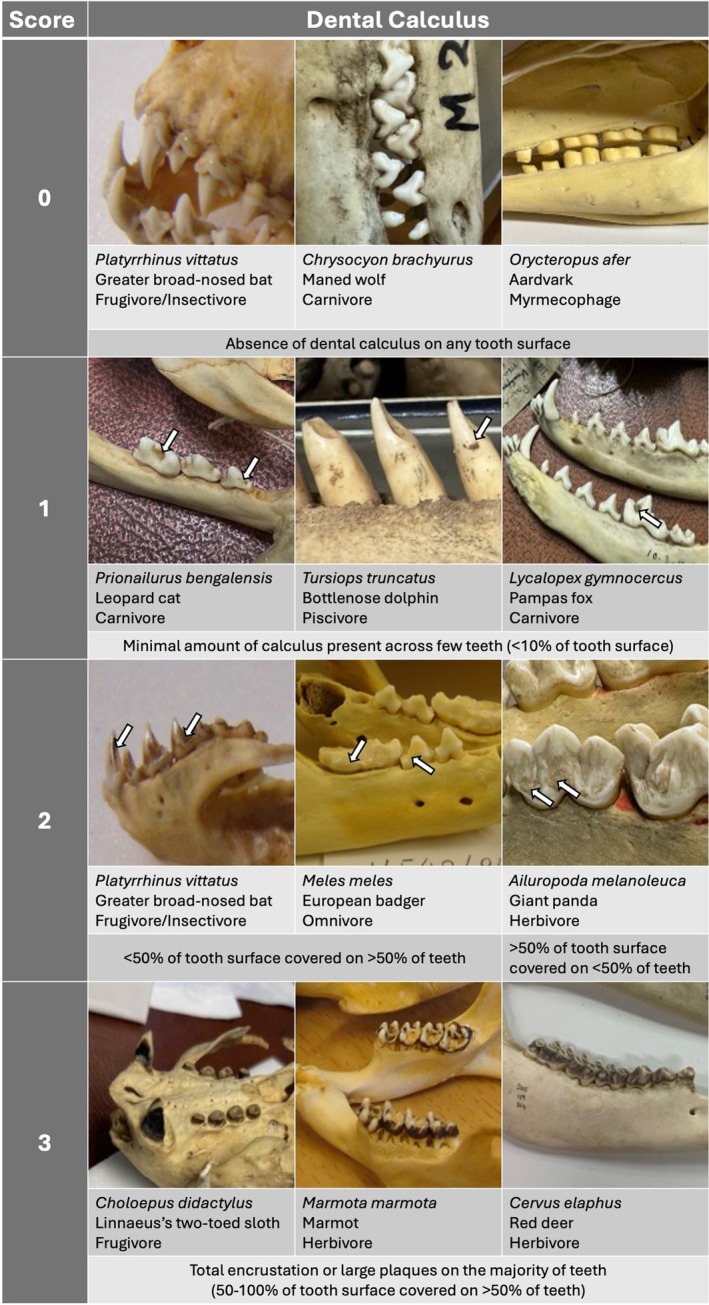
Examples of specimen dental calculus (DC) scores. DC scores given (on the left, 0–3) for 11 species of mammal. Common names and dietary classification according to Lintulaakso et al. (2023) are provided for each specimen. Arrows indicate calculus deposits. Two specimens of *P. vittatus* with different scores are shown.

We avoided specimens that showed obvious evidence of tooth cleaning, for example, excessive glue affixing teeth, polished bone, varnished tooth surfaces, etc., often associated with specimen preparation and handling. Captivity status was also taken from the labels; for the purposes of our analysis, we treated any indication of time spent in captivity as “captive”. Teeth were scored and classified according to the presence and abundance/amount of calculus (Figure 1), with 0 = no calculus; 1 = minimal amount of calculus present across few teeth (< 10% of tooth surface); 2 = several large plaque deposits across multiple teeth (> 50% of tooth surface covered on < 50% of teeth, e.g., Figure 1: Giant panda) or most teeth with similar low amount of calculus (< 50% of tooth surface covered on > 50% of teeth, e.g., Figure 1: European badger); 3 = total encrustation or large plaques on the majority of teeth (50%–100% of tooth surface covered on > 50% of teeth).

Teeth were scored separately for upper and lower jaws (maxillae and mandible), lingual and buccal tooth surface, and tooth type (incisor, canine, and [pre]molar) (see schematic tooth representation: Figure S1). We aimed to survey 10 specimens per species when possible, though due to collection limitations and missing teeth, some species were represented by fewer specimens (Table S1). When possible, we aimed to survey geographically and temporally diverse specimens to avoid a potential batch effect resulting from museum preparation practices or environment. Only teeth set in the jaw were surveyed to increase the confidence in tooth type assignment and specimen identity. Teeth not set in the jaw, even if showing a clear tooth type morphology and association with a specimen (e.g., in the same box), were disregarded. Odontoceti have a homodont tooth morphology, and these teeth were all classed as molars for the purpose of our analysis. Statistical and comparative analyses were carried out only on species for which four or more specimens were surveyed, for a total of 118 species, 110 genera, and 64 families, short only one order, Hyracoidea. However, the majority of species had 10 specimens surveyed (*n* = 72). The results of the survey, including species with fewer than four individuals, are presented in Table S1.

Using an ANOVA, we first tested if tooth type (incisor, canine, molar), jaw (upper or lower), and side (buccal or lingual) differed in how much dental calculus they showed (Figure S2A,B). To this end, we took averages of dental calculus measures for all other non‐focal variables. For example, to obtain a mean score per tooth type for each species, we averaged upper and lower lingual and buccal scores for the given tooth type across all individuals. Only the tooth type was significant (see Section 3) and we refer to the mean score per tooth type as DC score (Table S2). We used molar DC scores for all subsequent analyses, unless specified otherwise, as molars were the most consistently present tooth type in the study specimens and thus the datasets had the least amount of missing data (Table S1).

To test for differences in DC score related to diet, we assigned surveyed species into diet groups following the “calculated cluster mean diet” classification from Lintulaakso et al. (2023). We used an ANOVA to test for differences in molar DC score between the four diet groups: animalivore (*n* = 48), herbivore (*n* = 31), frugivore (*n* = 18), and omnivore (*n* = 21).

To rule out the possibility that different preservation approaches and specimen preparation techniques could have affected dental calculus prevalence and abundance in different museums, we selected five species with at least four surveyed specimens per museum in two different museums and tested for the consistency in DC scores between the museums for the same species using an ANOVA with species as a nested factor within museum. We included species with different diets: two animalivorous species, *Panthera leo* (*n* = 10 NHMUK, *n* = 9 NHM Vienna) and *Puma concolor* (*n* = 7 NHMUK, *n* = 6 NMS); two herbivorous species, *Capreolus capreolus* (*n* = 9 NHM Vienna, *n* = 9 NMS) and *Galeopterus variegatus* (*n* = 10 NHMUK, *n* = 4 NHM Vienna); and the frugivorous *Pteropus vampyrus* (*n* = 7 NHM Vienna, *n* = 10 NMS).

To assess the effects of captivity on DC formation, for 71 species we surveyed either captive or both captive and wild individuals, with a total of 466 specimens that had been held in captivity. We used ANOVAs with captivity status as a nested factor within diet to test for differences in DC score between captive (primarily from zoos) and wild individuals across diet groups.

### 2.2 Phylogenetic Analyses

We tested for phylogenetic signal in dental calculus formation across all tooth types with two approaches implemented in the R packages picante and phylosignal (Cowan et al. 2020; Keck et al. 2016). Since one of the approaches relies on binary data, we converted DC scores into presence/absence records by collapsing mean DC scores per tooth type ≤ 0.1 to 0 (absence of DC) and mean scores > 0.1 to 1 (presence of DC) (Table S2). The cut‐off corresponds to a single specimen out of 10 having a maximum DC score for a specific tooth type (e.g., molar) on each surface (buccal and lingual) and jaw (upper and lower), or all specimens consistently showing a DC score of 1 for a specific tooth type on each side and jaw. We downloaded the MCC mammalian consensus tree constructed with DNA‐only records from vertlife.org, which contains 4098 of the reported ~6000 mammalian species (Upham et al. 2019). Five species we surveyed were not present in this tree. For three of them ( *Anomalurus derbianus* , *Tupaia montana* , and *Oryzorictes tetradactylus* ) we used a congeneric species as representative ( *A. beecrofti* , *T. glis* , and *O. hova* ). One species ( *Elephantulus fuscipes* ) was excluded from analysis as two congeneric species were already present, and one monotypic species ( *Lavia frons* ) was represented by its closest relative ( *Megaderma spasma* ). Previous studies have shown that the genus node to species tip distance does not strongly affect phylogenetic metrics (Qian and Jin 2020), and so the substitution of missing species with congenerics is unlikely to substantially affect our phylogenetic analyses.

The first phylogenetic approach utilized net relatedness index (NRI) and nearest taxon index (NTI) (Kembel and Hubbell 2006). Both indices are measures of phylogenetic clustering or overdispersion and, as such, can be used as a measure of phylogenetic signal. The NRI is a measure of the difference in mean pairwise distance (MPD) between all species pairs with the trait present as compared to random reshuffling, and so indicates whether species with the trait are found more clustered than expected by chance. The NTI is similar but uses the mean nearest taxon distance (MNTD) between all species with the trait; the NTI shows if any two species with the trait are closer to each other than would be expected if the trait was randomly distributed.

Second, we used Pagel's lambda for the continuous trait values of mean DC score per tooth type (Gittleman and Kot 1990; Pagel 1999). Pagel's lambda is a measure of the transformation of a phylogeny that fits the trait data to a model of Brownian motion. A lambda value of 1 means the trait is distributed as expected under Brownian motion, and 0 is random (equivalent to a star phylogeny).

To assess potential bias in the phylogenetic representativeness of our sampling, we used NRI as a measure of phylogenetic clustering or overdispersion of surveyed genera. To do so, we collapsed the phylogeny to genus level, retaining one representative tip per genus. All genera in the tree were then scored with a binary classification of whether or not a species in that genus had been surveyed.

### 2.3 Diet Analysis

We assigned surveyed species into diet groups following the “calculated cluster mean diet” classification from Lintulaakso et al. (2023). To test for differences in molar DC score related to diet, we used an ANOVA for four diet groups: animalivore (*n* = 48), herbivore (*n* = 31), frugivore (*n* = 18), and omnivore (*n* = 21). The same publication was also used to obtain dietary nutritional composition for the surveyed mammalian species (Lintulaakso et al. 2023). This database lists diet nutrient composition per species divided into five components: crude fiber, inorganic residue (ash), crude fat (ether extract), protein, and sugars and starches (nitrogen‐free extract). We followed the tripartite dietary scheme in Lintulaakso et al. (2023), combining crude fiber and ash, crude protein and fats, and sugars and starches, roughly corresponding to herbivores, animalivores, and frugivores, respectively.

To disentangle the effects of diet and phylogeny on DC formation, we used a multilevel linear mixed effects model corrected for phylogenetic relatedness implemented in the R package Metafor (Viechtbauer 2010). We fitted diet nutritional composition as the explanatory variable and molar DC score as the response variable. As the diet data was compositional in nature (all nutrient components summed to 1), log‐ratios were used. Each of the three log‐ratios was used as a fixed predictor variable: Protein & Fats:Fiber; Sugars & Starches:Fiber; Protein & Fats:Sugars & Starches. To account for the effects of phylogeny, we included taxonomic species as a random effect and phylogeny as a variance–covariance matrix derived from the tree used above. To account for measurement errors, the squared standard error for each species' molar DC score was included as *V*.

### 2.4 Predictions of DC Scores Across the Mammalian Tree of Life

The model constructed above was used to make predictions of molar DC scores across ~4500 mammal species present in the diet composition database but not surveyed in this study. The species‐specific predicted DC scores were averaged by mammalian family to provide an estimate of the likelihood of dental calculus presence.

## 3 Results

### 3.1 Dental Calculus Is Present in Majority of Mammals

We identified DC as the presence of a visible deposit on the teeth of surveyed specimens that was clearly distinct from any tooth morphological features (Figure 1). The DC deposits varied in form, size, and color, ranging in appearance from chalky white plaques to a fine dark film. They were often confined to what was likely a supragingival position on the living individuals (Figure 1, examples for scores 2 and 3), supporting their identification as DC. A further confirmation for the identity of these varied deposits as DC comes from an associated study which sequenced samples from 403 individuals in 30 mammalian species and confirmed the presence of oral microbial taxa in all samples (Moraitou et al. 2025). We can thus be confident that here we report on dental calculus, the calcified dental plaque microbiome, from museum specimens (Figure S3).

**FIGURE 2:**
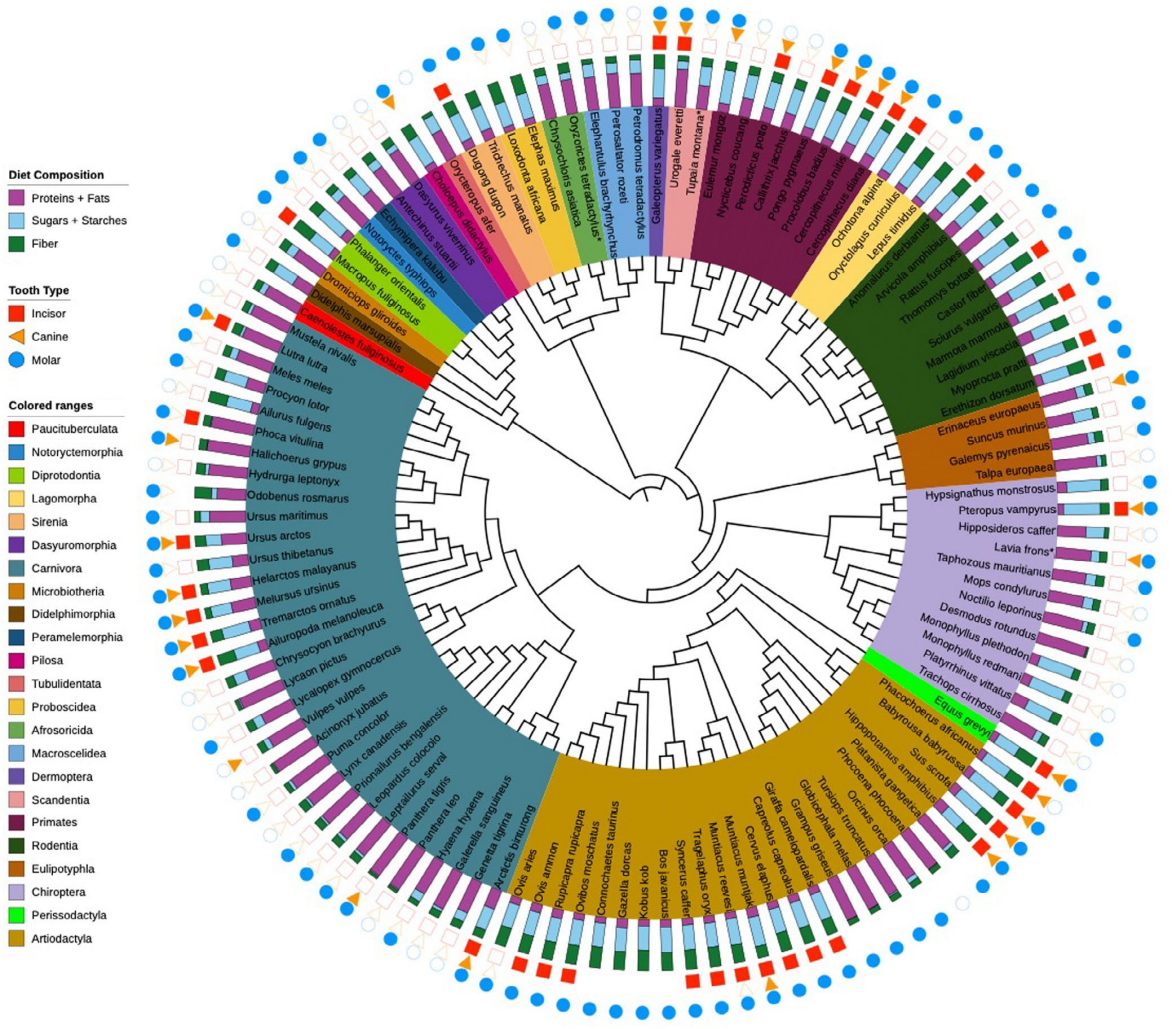
Dental calculus presence across the mammalian phylogeny and tooth types, showing species for which at least four wild specimens were available (*n* = 118). Mean scores per tooth type were binarized, with a score > 0.1 considered as DC presence and shown with a filled shape along the three outer rings. Unfilled shapes indicate a mean score ≤ 0.1, which was considered as absence of DC. No shape indicates that the tooth type was absent in the specimens surveyed or that the species lacks that tooth type altogether. All odontoceti teeth were scored as molars. This tree was downloaded from vertlife.org (Upham et al. 2019) using the MCC consensus tree of DNA‐only records. Asterisks indicate surveyed species not present in the downloaded tree. For *Anomalurus derbianus* , *Tupaia montana* , and *Oryzorictes tetradactylus* we used congeneric species as representative: *A. beecrofti* , *T. glis* , *O. hova* . Species are colored by mammalian order and dietary composition is shown as stacked bars.

**FIGURE 3:**
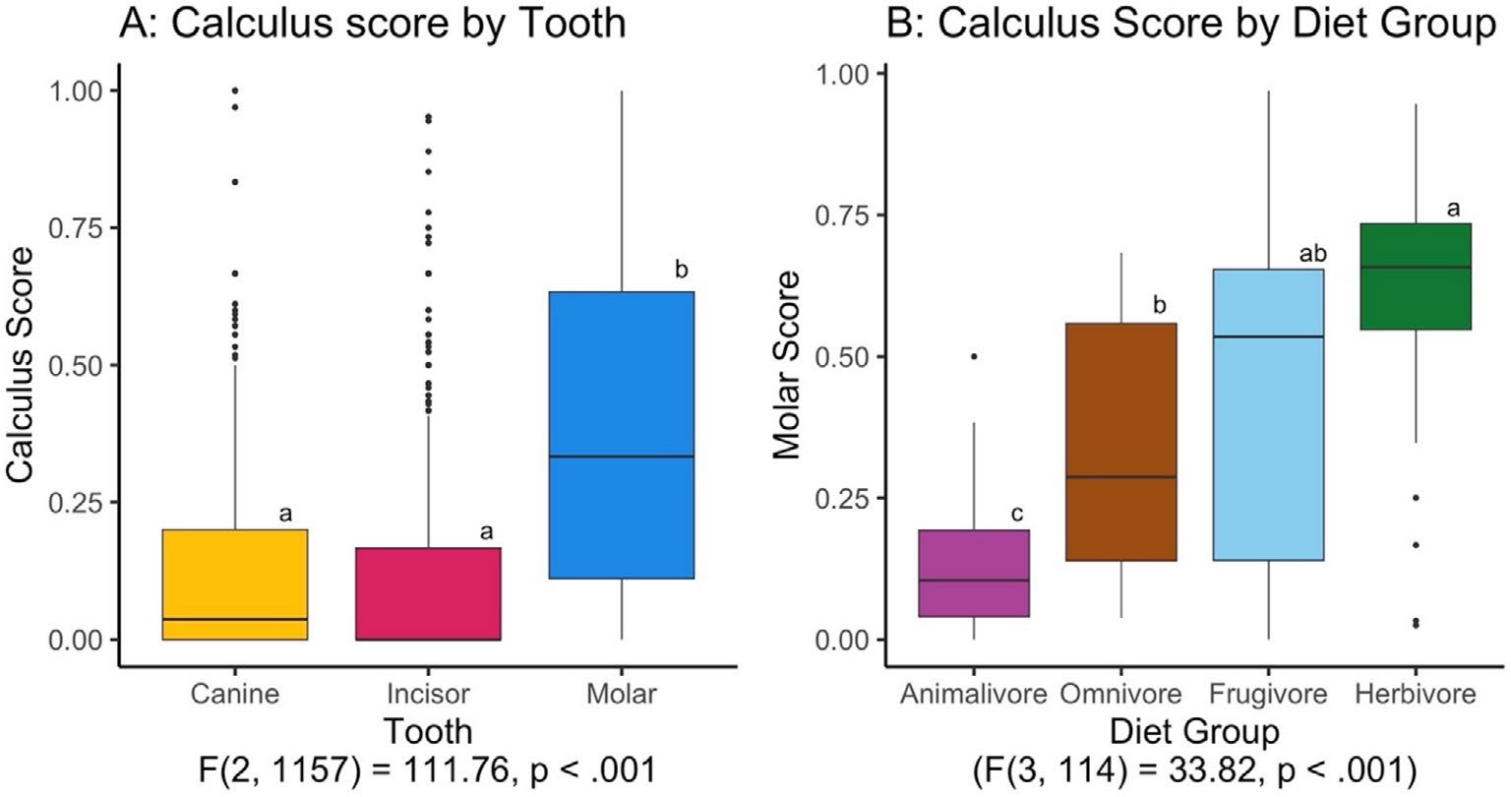
Comparisons of calculus scores by tooth type (A) and molar calculus scores by diet group (B). Scores for wild specimens only. Results from one‐way ANOVAs are shown below *x* axis label. Letters above the boxplots indicate results from post hoc Tukey's HSD tests. Values for post hoc tests (including *p*‐values corrected for multiple comparisons) can be found in Table S3.

We found no significant differences in DC scores for jaw (upper versus lower, *t*(1158) = −0.32, *p* = 0.748, Figure S2A) or side (lingual versus buccal, *t*(1156) = −0.60, *p* = 0.551, Figure S2B) within the entire dataset. However, the presence of calculus varied by tooth type. Specifically, molars most frequently and consistently showed DC deposits, so that if a species produced DC, it was present on the molars even if it was absent on other teeth (Figure 2). DC scores differed significantly across tooth types (ANOVA, *F*(2, 1157) = 111.76, *p* < 0.001), with molars consistently showing higher DC scores (Figure 3A). Post hoc Tukey's HSD tests showed that molars had significantly higher scores than both canines and incisors, but there was no significant difference between incisors and canines (Table S3A).

Additionally, molars were the most reliably present tooth type on the surveyed specimens, with many specimens missing incisors or canines but rarely missing molars (Table S1). Only three species did not follow this general pattern ( *Perodicticus potto* , *Vulpes vulpes* , and *Phacochoerus africanus* ), as they showed DC on incisors and/or canines, but not on molars (Figure 2, Table S1).

Of the 118 surveyed species with four or more specimens, 104 presented dental calculus, most commonly on the molars (Figure 2, Figure S4 for survey results including all 142 species, which were qualitatively similar). Calculus abundance varied within and across species (Table S1). Some species presented dental calculus in large accretions, regularly developing on the same tooth types. Other species consistently showed sparse calculus presence (Table S1). Few species showed absolutely no calculus (Figure 2, Figure S4). To evaluate if the obtained DC scores could be due to differences in specimen preservation and treatment among different natural history collections, we identified five species for which at least four specimens were surveyed in two different museums each. DC scores did not differ for four out of five species, whereas we observed a significant difference in DC scores for *Puma concolor* (Figure S2D). However, this difference may have an ecological explanation (see Section 4). In general, this result suggests that the observed DC scores are a biological feature of the surveyed species and not primarily influenced by preservation, treatment, and storage conditions in the museums.

### 3.2 Production of Dental Calculus Is Phylogenetically Constrained

The test for taxonomic representativeness of the surveyed genera showed that they were neither more nor less phylogenetically dispersed than random (NRI = −0.5, *p* = 0.7), supporting the absence of sampling bias in the species surveyed. Therefore, we next investigated any underlying phylogenetic structure to dental calculus formation. First, we used presence‐absence values for DC. NRI and NTI measure the effect size of MPD and MNTD, representing evolutionary relationships at a deep and shallow (tip to tip) level, respectively. NRI values of 1.96 and −1.96 have been used as thresholds beyond which traits are considered phylogenetically clustered or overdispersed, respectively (Coppi et al. 2019). For all tooth types, our results show clear evidence of clustering, with NRI values all statistically significant and > 2 (Table 1). No NTI values were significant, indicating that the distance from any species with DC to its nearest neighbor with DC was not different from what would be found if the trait was distributed randomly.

**TABLE 1:** Mean pairwise distance (MPD), mean nearest taxon distance (MNTD), net relatedness index (NRI), nearest taxon distance (NTI) values for all three tooth types.

Tooth		Observed	Random mean	Random SD	NRI, NTI
MOLAR (88/118)	MPD	165.45	176.87	5.61	2.03*
	MNTD	48.9	48.6	3.2	0.11
CANINE (28/82)	MPD	141.23	177.49	15.13	2.39*
	MNTD	61.00	74.04	9.60	1.35
INCISOR (39/108)	MPD	145.21	176.87	10.99	2.88*
	MNTD	56.13	63.06	6.60	1.05

*Note:* Numbers in parentheses indicate the number of species presenting dental calculus for each tooth type under the binarized scoring scheme out of the number of species with that tooth type. Column Observed shows values calculated from our data, Random Mean and Random SD show the mean and standard deviation from the null model of 999 random tip reshufflings. NRI and NTI measure phylogenetic clustering or overdispersion, equal to the standardized effect size multiplied by −1.

* *p* < 0.05.

Second, we used Pagel's lambda to test for the presence of phylogenetic signal in molar DC score. Pagel's lambda ranges from 0 to 1, with 1 representing an underlying phylogenetic structure following a model of evolution under Brownian motion. We recovered a value of 0.83 (*p* < 0.05) using species molar DC scores, confirming the presence of phylogenetic signal.

While we aimed to survey at least one species in every mammalian order to ensure phylogenetic/taxonomic breadth, some clades are better represented in collections, and consequently in our dataset, than others. Sensitivity analyses for the effect of species and family sampling show little to no effect on our conclusions about the phylogenetic signal of DC formation (< 5% effect on lambda value) (see Table S4).

### 3.3 Diet and Evolutionary Relationships Impact Dental Calculus Formation

DC formation is expected to be influenced by diet (Jin and Yip 2002; Lieverse 1999; Radini et al. 2017). We therefore considered if mammals belonging to different dietary groups differ in the build‐up of DC. Indeed, we found a significant difference in DC scores between dietary groups, with herbivores showing higher DC scores than animalivores and omnivores (*F*(3, 114) = 33.82, *p* < 0.001, Figure 3B). Frugivores, herbivores, and omnivores had significantly higher scores than animalivores in post hoc Tukey's HSD tests. The difference between herbivores and frugivores was not significant (*p* = 0.058) and similarly, omnivores did not significantly differ from frugivores (*p* = 0.529), but had lower scores than herbivores (Table S3B).

As captivity affects diet (among many other aspects), we also tested for differences in DC scores between wild and captive individuals in our dataset. Captivity had a significant effect on the DC scores (nested ANOVA, *F*(6, 856) = 57.03, *p* < 0.001, Figure S2C). Molar scores for captive individuals were more similar to each other across diet groups than for wild individuals (Figure S2C), suggesting a homogenizing effect of captivity. Specifically, average molar DC scores for species with animalivorous diets were higher in captivity than in the wild, whereas average molar DC scores for species with herbivorous and frugivorous diets were lower.

**FIGURE 4:**
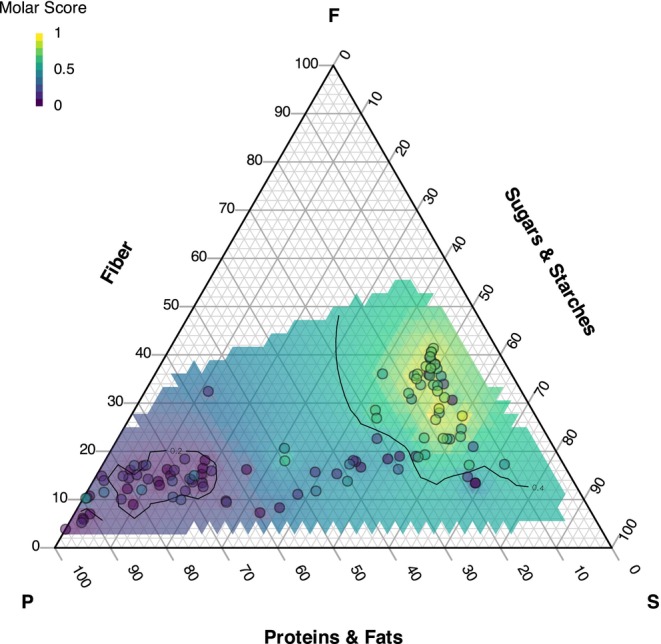
Ternary plot of molar calculus scores and diet nutritional composition. Each circle indicates one species included in this survey. The shaded area covers the full range of diet nutritional composition data taken from Lintulaakso et al. (2023). Letters in the corners indicate the point of maximum value for each nutritional component: F, fibers; S, sugars & starches; P, proteins and fats. The color of the area indicates the projected molar calculus score based on our survey results. Colored circles indicate the scores from surveyed species. Contour lines delineate areas in diet space with high (right) and low (left) expected DC formation based on diet nutritional composition.

We next examined which nutritional components affect DC formation. To this end, we used the tripartite dietary nutrition structure in Lintulaakso et al. (2023), reflecting the main diet groups: proteins and fats for animalivory, sugars and starches for frugivory, and fibers for herbivory. DC scores were highest for species with low protein and fat content in their diets (Figure 4), mirroring the analyses based on dietary group assignments above. However, the presence of outliers—species that did not form DC although their dietary composition is suitable for DC formation and vice versa—suggested that factors other than dietary nutritional components affect DC build‐up.

Diet is associated with morphological traits like tooth shape. Indeed, tooth shape has been used to infer diet (see Evans and Pineda‐Munoz 2018), and has been found to show some degree of phylogenetic signal (Reuter et al. 2023). Therefore, the phylogenetic signal we recovered for DC formation could directly reflect dietary nutritional composition but also other phylogenetically structured factors, such as oral and digestive tract morphology and physiology. We aimed to separate the factors leading to DC formation resulting from the nutritional components of diet and those resulting from other biological/ecological variables that may also have an underlying phylogenetic structure. To achieve this, we constructed a multilevel linear mixed‐effects model that takes both host phylogeny and dietary nutritional composition into account. The model returned a much stronger effect of protein‐to‐fiber ratio than protein‐to‐sugars ratio, with the fibers‐to‐sugars ratio removed as its effect was redundant and negligible (Table 2). After accounting for the effect of diet on DC formation, the remaining variance was split approximately evenly between taxonomic species and phylogeny, reflecting shallow and deep evolutionary scales, respectively (Table 2).

**TABLE 2:** Phylogenetically‐informed diet model.

Variance components	Proportion
Phylogeny	0.54
Taxonomic species	0.46

Log‐ratios	Estimate	Std. error	*p*
Protein:Fiber	**−1.7**	**0.29**	**< 0.001**
Protein:Sugars	0.07	0.1	0.43

*Note:* Log ratios were used to compare the effects of dietary nutrient proportion. The effect of fibers: Sugars was negligible and redundant. Variance components Phylogeny and Taxonomic Species were included as random effects of a phylogenetic distance matrix and phylogeny‐agnostic taxonomic groups, respectively.

### 3.4 Dental Calculus Predictions

**FIGURE 5:**
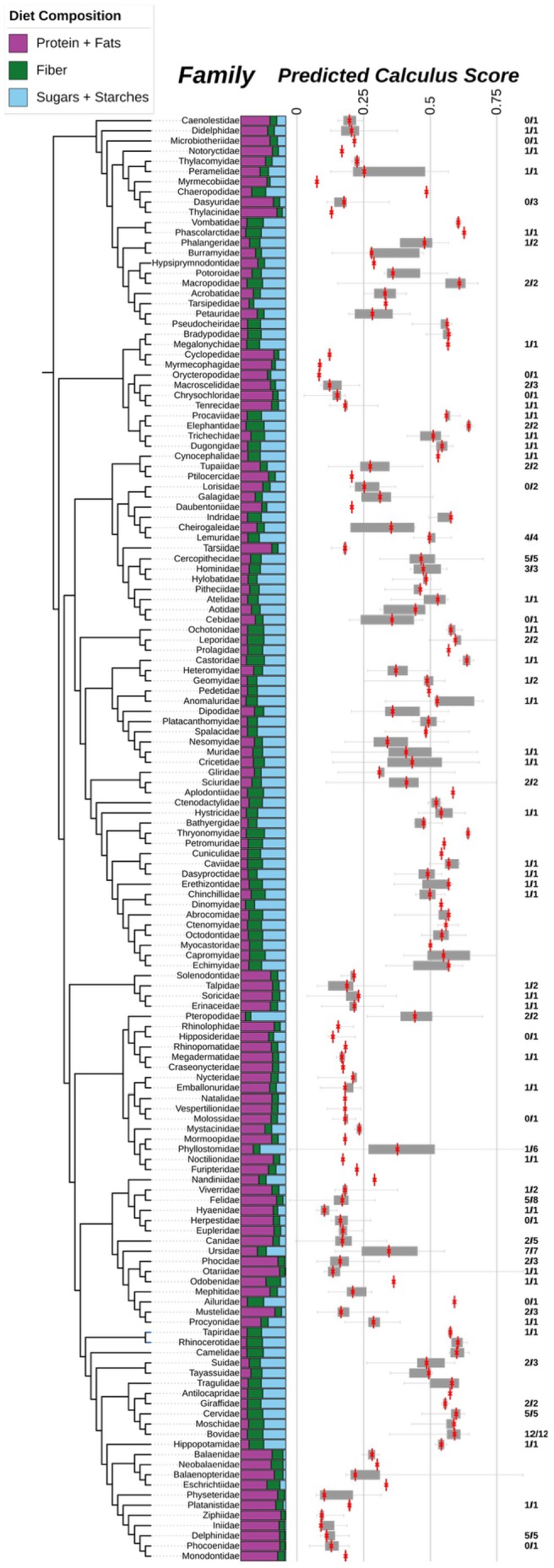
Predicted molar calculus scores by mammalian family. Phylogenetic tree of mammalian families, showing mean diet compositions (bars) and predicted calculus scores (boxplots) per family. Mean value per family indicated by red bars. Fractions to the right of the boxplot relate to the species surveyed in this study, showing the number of species with calculus out of the total number of species surveyed in a given family. The tree was downloaded from vertlife.org (Upham et al. 2019) using the MCC consensus tree of DNA‐only records.

To facilitate oral microbiome research across the diversity of mammals, we used the insights obtained here to make predictions about the presence and abundance of dental calculus across the mammalian tree of life. Using the above model (Table 2) and dietary information from Lintulaakso et al. (2023), we made predictions about dental calculus scores for ca. 4500 mammal species that were not included in the survey (Table S5). These predictions were then combined, taking the mean molar DC score value per mammalian family to provide indications for clades that are most likely to present calculus (Figure 5).

## 4 Discussion

### 4.1 Dental Calculus Survey

Although the use of DC in addressing ecological and evolutionary questions of (historical) oral microbiota and host species is increasing, it has so far been limited to only a few mammalian species, with the focus on primates (Brealey et al. 2021; Fellows Yates et al. 2021; Moraitou et al. 2022; Ozga and Ottoni 2023; Power, Salazar‐García, Wittig, et al. 2015). Our survey across more than 1600 wild and captive specimens in five natural history museums demonstrated that DC is present in a large diversity of mammals, representing all major dietary groups, life histories, and habitats. By scoring DC presence and abundance, we revealed some general rules for the development of this calcified oral microbiome in mammals.

We found that DC was most reliably present on molars, even though many species also showed calculus deposits on incisors and canines. Molars were the most abundant tooth type retained in the jaw of dried preserved specimens in natural history collections, enabling reliable association between the individual specimen and tooth. Additionally, molars are used for chewing as opposed to biting or tearing, and their position in the mouth may allow for saliva and particulate matter to accumulate and remain in their vicinity for longer than for other tooth types, potentially promoting dental calculus build‐up.

Specimen cleaning and preparation practices certainly varied widely from institution to institution and through time, as methods and the human ideal of a perfect specimen changed. Unfortunately, records on how individual specimens were prepared are generally rarely available, making it extremely difficult to evaluate how different practices may have affected retention of dental calculus on the specimen teeth. Past accidental calculus removal in some specimens is also almost guaranteed, considering decades of handling, cleaning, sampling, moving, and manipulation. Our specimen selection attempted to account for it by avoiding specimens with obvious signs of cleaning. However, for four of five species for which we had four or more specimens in at least two different museums (species which included representatives of every diet group), we found no significant difference in calculus scores, indicating that differences in specimen treatment and preparation among museums were not impacting the results of our survey. The only exception to this was *Puma concolor* (Figure S2D). This species has a large geographic range covering the Americas. The majority of samples from this species surveyed at NHMUK (six of seven) were from South America, whereas the majority of samples at NMS (four of six) were from North America, corresponding to two different subspecies living under different ecological conditions (Culver 2000). Therefore, although differences in specimen treatment and preservation cannot be excluded, there could be a biological explanation for the observed differences in DC scores. Further, in a species with a virtually identical dietary nutritional composition ( *Panthera leo* , see diet composition in Table S2), we found no significant differences between museums (Figure S2D). We highlight the possibility that differences in ecology, as a result of geographic range, or age of the individual could contribute to the observed differences in *Puma*. While we acknowledge that preparation methods, storage conditions, and handling routines could result in variation in DC scores across museums, we generally observed good correspondence in DC scores from different collections.

We fully acknowledge that factors such as age, sex, or individual life history may affect calculus formation, as for each host taxon, diet may differ by age, sex, or local conditions (Birks and Dunstone 1985; Harrison 1983; Naganuma et al. 2020). Age information is generally rarely recorded for museum specimens. Only 140 of the wild specimens we surveyed had any information on age. Even when recorded, broad age classifications such as “adult” can encompass many years, over which calculus development may be affected. Future studies, targeting specific host taxa, could benefit from more detailed age estimates and enable comparisons across individuals of the same age class, sex, and life history. At the broad taxonomic range of the present study, we were limited by the information available in the metadata associated with each specimen (Table S1). In general, we selected specimens with complete tooth eruption so that the majority of surveyed specimens were adults (Table S1). Adult individuals are most commonly collected and present in museum collections, as has previously been reported (Holmes et al. 2016).

### 4.2 Diet and Phylogeny Explain DC Formation

Comparing DC formation between diet groups, we found that herbivores and frugivores consistently developed more calculus than animalivores. This is in line with expectations based on the literature, with carbohydrates and starches frequently reported as contributing to DC formation (Akcalı and Lang 2018; Lieverse 1999).

We found that captivity strongly affected DC abundance, with DC scores being more similar among captive individuals belonging to different dietary groups than among wild individuals. We followed the captivity designations from specimen labels. While time in captivity may have varied, we chose to consider any evidence of time in captivity as potentially influencing diet and hence DC formation. Captivity most strongly affected DC formation in species with animalivorous diets, with captive individuals showing higher DC scores and hence more calculus than wild individuals (Figure S2C). Processed diets, often supplemented with commercial pet food fed to big cats in captivity, have been shown to have higher carbohydrate content compared to wild whole‐carcass diets (Dzierga 2014; Kapoor et al. 2016; Kerr et al. 2013; Whitehouse‐Tedd et al. 2015), which likely leads to a greater development of DC. On the other hand, herbivores and frugivores both showed decreases in DC formation in captivity, likely due to supplemental crude protein and fat in their feeds (often exceeding 30% in total) leading to reduced calculus formation (Fens and Marcus Clauss 2024). Mechanical properties of food in captivity also likely have an effect on DC formation. Previous studies in animalivores have shown that softer diets can lead to plaque build‐up and disease, as captive diets lack grit that abrade teeth during mastication (Crates et al. 2023; O'Regan and Kitchener 2005). While captive individuals often live longer than wild individuals (Tidière et al. 2016), and this extended lifespan may have some additional effect on DC formation, our observations of differential effects of captivity on host species with different diets suggest that diet likely overrides the effects of longer lifespan.

Looking in detail at specific nutrients, as opposed to broad dietary groups, most previous studies suggest that carbohydrates are the main factor contributing to calculus formation (Buckley et al. 2014; Radini et al. 2017), as they provide the substrate for the salivary amylase that breaks down sugars in the saliva and enables formation of a film on the hard tooth surface, to which bacteria may adhere. In line with this, we found the highest rates of DC formation in species with a diet consisting of > 70% fibers, sugars, and starches (Figure 4, right contour bound). Species with high‐protein diets (Figure 4, left contour bound) were much less likely to develop dental calculus.

However, the formation of DC is dependent on more than just dietary starches, and indeed we identified many outliers (Figure 4). Our results indicate that non‐dietary factors may contribute to DC formation. We detected significant phylogenetic clustering at a deeper taxonomic scale but not at the tree tips. This indicates that while there is an underlying phylogenetic structure to DC formation, a given species with calculus is not more likely than random to be sister to another species that also forms calculus. Phylogenetic signal was also detected using continuous DC values. Following this, in the modeling we chose to include taxonomic species and phylogenetic species to represent differences in the phylogenetic depth of the DC formation. Similar to the NRI versus NTI, phylogenetic species refers to the deeper scale of tree‐wide phylogenetic distances (quantified by the variance–covariance matrix derived from the phylogenetic tree), while taxonomic species treats each species as a discrete group, agnostic of the degree of their phylogenetic relatedness. We found that the variance in our model was split approximately evenly between these two levels, indicating that while there is phylogenetic structure to how the traits evolved, closely related species in our survey may differ in calculus formation from their closest relatives, possibly resulting from recent dietary changes. This result is exemplified in the binturong, polar bear, and spiny bandicoot (Figure S5, *Arctictis binturong* , *Ursus maritimus* , *Echymipera kalubu* ), which all show a shift in both diet and calculus formation compared to their closest relative.

Other phylogenetically structured morphological factors may play a role in DC formation. Both the NRI and our modeling show phylogenetic signal at a deeper level, such as that between orders, partially explaining patterns in DC formation. The biological and ecological factors that contribute to this are likely as varied as mammalian oral physiology itself (Hernández‐Castañeda et al. 2015; Lieverse 1999).

Carnivores and aquatic mammals have the smallest salivary glands; seals have very small salivary glands and no parotid salivary glands (Tucker 1958). Development of the glands varies as well, with large parotid glands common in herbivores (Hofmann et al. 2008). The parotid gland is the largest and most important salivary gland in ruminants, providing half the salivary flow (Kay 1987). In most ruminants (including several species we surveyed), the opening of the parotid salivary duct is located opposite the first upper molar (Hofmann et al. 2008; Kay 1987; Nourinezhad et al. 2021), potentially accounting for the high levels of calculus encrustation on the upper jaws of bovids and other ruminants. Although we did not detect significant differences in DC scores between lower and upper jaw when considering all species, in bovids we found higher molar DC scores on the upper jaw compared to the lower jaw (Figure S6A).

While we focused our analysis primarily on molars because incisors and canines were frequently missing, differences in tooth morphology most certainly affect dental calculus formation. As an example, rodents and lagomorphs have a diet clearly amenable to dental calculus formation. The molars in these mammalian orders consistently showed high levels of encrustation (Figure S6B). However, calculus was rarely found on incisors. Both rodents and lagomorphs possess continuously growing incisors, which likely prevents DC formation through abrasion and loss.

### 4.3 Practical Considerations and Future Perspectives

Shifting priorities and trends in museums' philosophies of collecting or decisions about what to collect and how to preserve specimens made by curators in the past undoubtedly have an effect on the calculus that is preserved today, much as the choices made by curators today will impact research in the years to come. The natural history collections started and amassed largely in Western Europe in the 19th century resulted from a time when scientific priorities lay in exploration and cataloging (Holmes et al. 2016; Lister 2011; Schindel and Cook 2018; Winker 2004). While these collections have been extensively used for many questions of morphology‐based taxonomy and systematics, as originally intended, technological developments have repeatedly opened up new avenues of inquiry and expanded the possibilities of what can be done with museum collections (Bein et al. 2025; Malakasi et al. 2019; Nolen et al. 2024; Winker 2004; Yeates et al. 2016). Long‐term studies on ecological, evolutionary, and environmental change are especially critical in a time of climate change and biodiversity loss, and historical collections can provide a valuable glimpse into the past, preserving what may be under threat today (Bi et al. 2013; Brealey et al. 2021; Hahn et al. 2020; Nakahama 2021; Van Der Valk et al. 2019; Yeates et al. 2016).

Collections, with forward‐looking perspectives such as the continued acquisition of contemporary specimens, or a holistic understanding of what constitutes a specimen, inclusive of its parasites, microbiota, environment, etc. (Lendemer et al. 2020; Spellman 2019) will one day serve future research in ways perhaps unanticipated today (see “The Next Generation of Natural History Collections”, Schindel and Cook 2018). Specimen preparation practices can also be rethought to minimize cleaning and maximize preservation of DC, or keeping future sampling in mind when acquiring new specimens, such as prioritizing easy‐to‐sample lower jaws rather than larger, heavy skulls/upper jaws in ungulates.

Our results show that DC is found in a large diversity of mammals, is primarily present on molars, and generally does not differ in abundance between jaws or tooth sides. Because ease and efficiency of sampling differ between the upper and lower jaws and inner and outer tooth surfaces, these aspects, along with curators' interests, must be taken into consideration when designing future studies. For most larger species, particularly ungulates, lower jaws are easily handled, frequently collected in large‐scale, long‐term studies, and can provide a sufficient amount of calculus from one or two teeth (Moraitou et al. 2025). Smaller species, while potentially developing large amounts of dental calculus, may not be as easy to sample due to the small size of the skull and fragility of teeth and bones. In addition to historical collections, archaeozoological collections can also be sources of DC from much more distant times. Teeth can survive many taphonomic processes and are often found in abundance in excavations, presenting calculus deposits suitable for sampling (see DC on iron age cattle, Figure S7).

Using general rules of DC formation that are based on host taxonomy and dietary composition, as derived from our study dataset, we generated predictions across a large number of mammalian species. Our aim is to facilitate research planning for future researchers and projects, and to encourage and inform the use of dental calculus from museum collections. Our predictions show that most mammalian families have an average predicted DC score > 0.25. In our previous study, we found species with a score > 0.5 to be well suitable for sampling and that roughly 0.8 mg of DC material is sufficient for metagenomic sequencing and analyses (Moraitou et al. 2025). Species with average scores between 0.2 and 0.5 could still yield a suitable amount of calculus, but with lower amounts of encrustation, tooth size and the number of specimens available will have a greater influence on the possibility of collecting enough material. Even species with high levels of encrustation may not be suitable for sampling due to their small size (which may require pooling of multiple individuals) or distinctive skull/oral morphology.

Our DC predictions can help inform project planning for researchers interested in (preserved) oral microbiomes, by giving an indication of species that most reliably produce dental calculus. Phytoliths, starch grains, and other materials can also be recovered from DC and there is much value in the use of DC for more traditional (i.e., non‐genomic) archaeological and biological research. To help with that, we produced a visual guide for the identification of dental calculus in diverse mammals (Figure S8). Museum curators can also benefit from anticipating which parts of the collection may be of interest to researchers, and help inform collection and acquisition priorities with the future value of DC in mind.

## Author Contributions

**John L. Richards:** conceptualization (equal), data curation (equal), formal analysis (lead), investigation (lead), methodology (lead), writing – original draft (lead), writing – review and editing (lead). **Markella Moraitou:** conceptualization (equal), data curation (equal), investigation (equal), methodology (equal). **Ella Nates:** data curation (supporting), investigation (supporting). **Konstantina Saliari:** data curation (supporting), resources (equal), visualization (supporting), writing – review and editing (supporting). **Emmanuel Gilissen:** data curation (supporting), resources (equal), writing – review and editing (supporting). **Zena Timmons:** data curation (supporting), resources (equal), writing – review and editing (supporting). **Andrew C. Kitchener:** data curation (supporting), resources (equal), resources (equal), writing – review and editing (supporting), writing – review and editing (supporting). **Olivier S. G. Pauwels:** data curation (supporting), resources (equal), writing – review and editing (supporting). **Richard Sabin:** data curation (supporting), resources (equal), writing – review and editing (supporting). **Phaedra Kokkini:** data curation (supporting), resources (equal), writing – review and editing (supporting). **Roberto Portela Miguez:** data curation (supporting), resources (equal), writing – review and editing (supporting). **Katerina Guschanski:** conceptualization (equal), funding acquisition (lead), investigation (supporting), methodology (supporting), project administration (lead), supervision (lead), writing – review and editing (equal).

## Funding

This work was supported by the Swedish Research Council (Formas) grant 2019‐00275 and a studentship from the Darwin Trust of Edinburgh (SC006400).

## Conflicts of Interest

The authors declare no conflicts of interest.

## Supporting information


**Figure S1:** ece374105‐sup‐0001‐FiguresS1‐S8.docx.

**Table S1:** ece374105‐sup‐0002‐TablesS1‐S5.xlsx.

## Data Availability

All survey data, including specimen metadata and museum accession numbers, surveyed species calculus scores, statistical analysis summary tables, and predictive modeling output are available in the Supporting Information.

## References

[ece374105-bib-0001] Adler, C. J. , K. Dobney , L. S. Weyrich , et al. 2013. “Sequencing Ancient Calcified Dental Plaque Shows Changes in Oral Microbiota With Dietary Shifts of the Neolithic and Industrial Revolutions.” Nature Genetics 45: 450–455. 10.1038/ng.2536. 23416520 PMC3996550

[ece374105-bib-0002] Akcalı, A. , and N. P. Lang . 2018. “Dental Calculus: The Calcified Biofilm and Its Role in Disease Development.” Periodontology 2000 76, no. 1: 109–115. 10.1111/prd.12151. 29194797

[ece374105-bib-0003] Armitage, P. L. 1975. “The Extraction and Identification of Opal Phytoliths From the Teeth of Ungulates.” Journal of Archaeological Science 2, no. 3: 187–197. 10.1016/0305-4403(75)90056-4.

[ece374105-bib-0004] Bein, B. , I. Chrysostomakis , L. S. Arantes , et al. 2025. “Long‐Read Sequencing and Genome Assembly of Natural History Collection Samples and Challenging Specimens.” Genome Biology 26, no. 1: 25. 10.1186/s13059-025-03487-9. 39930463 PMC11809032

[ece374105-bib-0005] Bi, K. , T. Linderoth , D. Vanderpool , J. M. Good , R. Nielsen , and C. Moritz . 2013. “Unlocking the Vault: Next‐Generation Museum Population Genomics.” Molecular Ecology 22, no. 24: 6018–6032. 10.1111/mec.12516. 24118668 PMC4134471

[ece374105-bib-0006] Birks, J. D. S. , and N. Dunstone . 1985. “Sex‐Related Differences in the Diet of the Mink *Mustela vison* .” Ecography 8, no. 4: 245–252. 10.1111/j.1600-0587.1985.tb01175.x.

[ece374105-bib-0007] Brealey, J. C. , H. G. Leitão , T. Hofstede , D. C. Kalthoff , and K. Guschanski . 2021. “The Oral Microbiota of Wild Bears in Sweden Reflects the History of Antibiotic Use by Humans.” Current Biology 31, no. 20: 4650–4658.e6. 10.1016/j.cub.2021.08.010. 34437844

[ece374105-bib-0008] Brealey, J. C. , H. G. Leitão , T. van der Valk , et al. 2020. “Dental Calculus as a Tool to Study the Evolution of the Mammalian Oral Microbiome.” Molecular Biology and Evolution 37, no. 10: 3003–3022. 10.1093/molbev/msaa135. 32467975 PMC7530607

[ece374105-bib-0009] Buckley, S. , D. Usai , T. Jakob , A. Radini , and K. Hardy . 2014. “Dental Calculus Reveals Unique Insights Into Food Items, Cooking and Plant Processing in Prehistoric Central Sudan.” PLoS One 9, no. 7: e100808. 10.1371/journal.pone.0100808. 25028938 PMC4100759

[ece374105-bib-0010] Ciancio, M. R. , E. C. Vieytes , M. C. Castro , and A. A. Carlini . 2021. “Dental Enamel Structure in Long‐Nosed Armadillos (Xenarthra: Dasypus) and Its Evolutionary Implications.” Zoological Journal of the Linnean Society 192, no. 4: 1237–1252. 10.1093/zoolinnean/zlaa119.

[ece374105-bib-0011] Ciochon, R. L. , D. R. Piperno , and R. G. Thompson . 1990. “Opal Phytoliths Found on the Teeth of the Extinct Ape Gigantopithecus Blacki: Implications for Paleodietary Studies.” Proceedings of the National Academy of Sciences 87, no. 20: 8120–8124. 10.1073/pnas.87.20.8120. PMC54904 2236026

[ece374105-bib-0012] Clarke, D. , and A. Cameron . 1998. “Relationship Between Diet, Dental Calculus and Periodontal Disease in Domestic and Feral Cats in Australia.” Australian Veterinary Journal 76, no. 10: 690–693. 10.1111/j.1751-0813.1998.tb12284.x. 9830570

[ece374105-bib-0013] Coppi, A. , L. Lazzaro , E. Ampoorter , L. Baeten , K. Verheyen , and F. Selvi . 2019. “Understorey Phylogenetic Diversity in Thermophilous Deciduous Forests: Overstorey Species Identity Can Matter More Than Species Richness.” Forest Ecosystems 6, no. 1: 37. 10.1186/s40663-019-0191-1.

[ece374105-bib-0014] Cowan, P. D. , M. R. Hel‐ , H. Morlon , O. Webb , and M. S. W. Kembel . 2020. “Package ‘picante’.” 10.1093/bioinformatics/btq166>.License.

[ece374105-bib-0015] Crates, R. , D. Stojanovic , and R. Heinsohn . 2023. “The Phenotypic Costs of Captivity.” Biological Reviews 98, no. 2: 434–449. 10.1111/brv.12913. 36341701

[ece374105-bib-0016] Culver, M. 2000. “Genomic Ancestry of the American Puma ( *Puma concolor* ).” Journal of Heredity 91, no. 3: 186–197. 10.1093/jhered/91.3.186. 10833043

[ece374105-bib-0017] De La Fuente, C. , S. Flores , and M. Moraga . 2013. “Dna From Human Ancient Bacteria: A Novel Source of Genetic Evidence From Archaeological Dental Calculus.” Archaeometry 55, no. 4: 767–778. 10.1111/j.1475-4754.2012.00707.x.

[ece374105-bib-0018] Dzierga, B. M. 2014. “Zutrition: Analyzing and Evaluating Diets Fed to Captive Mammals at Capron Park Zoo.”

[ece374105-bib-0019] Evans, A. R. , and S. Pineda‐Munoz . 2018. “Inferring Mammal Dietary Ecology From Dental Morphology.” In Methods in Paleoecology: Reconstructing Cenozoic Terrestrial Environments and Ecological Communities, edited by D. A. Croft , D. F. Su , and S. W. Simpson , 37–51. Springer International Publishing. 10.1007/978-3-319-94265-0_4.

[ece374105-bib-0020] Fellows Yates, J. A. , I. M. Velsko , F. Aron , et al. 2021. “The Evolution and Changing Ecology of the African Hominid Oral Microbiome.” Proceedings of the National Academy of Sciences of the United States of America 118, no. 20: e2021655118. 10.1073/pnas.2021655118. 33972424 PMC8157933

[ece374105-bib-0021] Fens, A. , and M. C. Marcus Clauss . 2024. “Nutrition as an Integral Part of Behavioural Management of Zoo Animals.” Journal of Zoo and Aquarium Research 12, no. 4: 196–204. 10.19227/jzar.v12i4.786.

[ece374105-bib-0022] Gittleman, J. L. , and M. Kot . 1990. “Adaptation: Statistics and a Null Model for Estimating Phylogenetic Effects.” Systematic Biology 39, no. 3: 227–241. 10.2307/2992183.

[ece374105-bib-0023] Hahn, E. E. , A. Grealy , M. Alexander , and C. E. Holleley . 2020. “Museum Epigenomics: Charting the Future by Unlocking the Past.” Trends in Ecology & Evolution 35, no. 4: 295–300. 10.1016/j.tree.2019.12.005. 31955919

[ece374105-bib-0024] Harrison, M. J. S. 1983. “Age and Sex Differences in the Diet and Feeding Strategies of the Green Monkey, Cercopithecus Sabaeus.” Animal Behaviour 31, no. 4: 969–977. 10.1016/S0003-3472(83)80001-3.

[ece374105-bib-0025] Hendy, J. , C. Warinner , A. Bouwman , et al. 2018. “Proteomic Evidence of Dietary Sources in Ancient Dental Calculus.” Proceedings of the Royal Society B: Biological Sciences 285, no. 1883: 20180977. 10.1098/rspb.2018.0977. PMC6083251 30051838

[ece374105-bib-0026] Hernández‐Castañeda, A. A. , G. C. Aranzazu‐Moya , G. M. Mora , and D. d. P. Queluz . 2015. “Chemical Salivary Composition and Its Relationship With Periodontal Disease and Dental Calculus.” Brazilian Journal of Oral Sciences 14: 159–165. 10.1590/1677-3225v14n2a12.

[ece374105-bib-0027] Hofmann, R. R. , W. J. Streich , J. Fickel , J. Hummel , and M. Clauss . 2008. “Convergent Evolution in Feeding Types: Salivary Gland Mass Differences in Wild Ruminant Species.” Journal of Morphology 269, no. 2: 240–257. 10.1002/jmor.10580. 17957712

[ece374105-bib-0028] Holmes, M. W. , T. T. Hammond , G. O. U. Wogan , et al. 2016. “Natural History Collections as Windows on Evolutionary Processes.” Molecular Ecology 25, no. 4: 864–881. 10.1111/mec.13529. 26757135 PMC4755843

[ece374105-bib-0029] Jin, Y. , and H.‐K. Yip . 2002. “Supragingival Calculus: Formation and Control.” Critical Reviews in Oral Biology & Medicine 13: 426–441. 12393761 10.1177/154411130201300506

[ece374105-bib-0030] Kapoor, V. , T. Antonelli , J. A. Parkinson , and A. Hartstone‐Rose . 2016. “Oral Health Correlates of Captivity.” Research in Veterinary Science 107: 213–219. 10.1016/j.rvsc.2016.06.009. 27473998

[ece374105-bib-0031] Kay, R. N. B. 1987. “Weights of Salivary Glands in Some Ruminant Animals.” Journal of Zoology 211, no. 3: 431–436. 10.1111/j.1469-7998.1987.tb01544.x.

[ece374105-bib-0032] Keck, F. , F. Rimet , A. Bouchez , and A. Franc . 2016. “Phylosignal: An R Package to Measure, Test, and Explore the Phylogenetic Signal.” Ecology and Evolution 6, no. 9: 2774–2780. 10.1002/ece3.2051. 27066252 PMC4799788

[ece374105-bib-0033] Kembel, S. W. , and S. P. Hubbell . 2006. “The Phylogenetic Structure of a Neotropical Forest Tree Community.” Ecology 87, no. sp7: S86–S99. 10.1890/0012-9658(2006)87[86:tpsoan]2.0.co;2. 16922305

[ece374105-bib-0034] Kerr, K. R. , C. L. Morris , S. L. Burke , and K. S. Swanson . 2013. “Apparent Total Tract Macronutrient and Energy Digestibility of 1‐to‐3‐Day‐Old Whole Chicks, Adult Ground Chicken, and Extruded and Canned Chicken‐Based Diets in African Wildcats ( *Felis silvestris lybica* ).” Zoo Biology 32, no. 5: 510–517. 10.1002/zoo.21084. 23818436

[ece374105-bib-0035] Lendemer, J. , B. Thiers , A. K. Monfils , et al. 2020. “The Extended Specimen Network: A Strategy to Enhance US Biodiversity Collections, Promote Research and Education.” Bioscience 70, no. 1: 23–30. 10.1093/biosci/biz140. 31949317 PMC6956879

[ece374105-bib-0036] Lieverse, A. R. 1999. “Diet and the Aetiology of Dental Calculus.” International Journal of Osteoarchaeology 9, no. 4: 219–232. 10.1002/(SICI)1099-1212(199907/08)9:4<219::AID-OA475>3.0.CO;2-V.

[ece374105-bib-0037] Lintulaakso, K. , N. Tatti , and I. Žliobaitė . 2023. “Quantifying Mammalian Diets.” Mammalian Biology 103, no. 1: 53–67. 10.1007/s42991-022-00323-6.

[ece374105-bib-0038] Lister, A. M. 2011. “Natural History Collections as Sources of Long‐Term Datasets.” Trends in Ecology & Evolution 26, no. 4: 153–154. 10.1016/j.tree.2010.12.009. 21255862

[ece374105-bib-0039] Mackie, M. , J. Hendy , A. D. Lowe , et al. 2017. “Preservation of the Metaproteome: Variability of Protein Preservation in Ancient Dental Calculus.” STAR: Science & Technology of Archaeological Research 3, no. 1: 58–70. 10.1080/20548923.2017.1361629. PMC5633013 29098079

[ece374105-bib-0040] Malakasi, P. , S. Bellot , R. Dee , and O. M. Grace . 2019. “Museomics Clarifies the Classification of Aloidendron (Asphodelaceae), the Iconic African Tree Aloes.” Frontiers in Plant Science 10: 1–11. 10.3389/fpls.2019.01227. 31681358 PMC6803536

[ece374105-bib-0041] Mann, A. E. , S. Sabin , K. Ziesemer , et al. 2018. “Differential Preservation of Endogenous Human and Microbial DNA in Dental Calculus and Dentin.” Scientific Reports 8, no. 1: 9822. 10.1038/s41598-018-28091-9. 29959351 PMC6026117

[ece374105-bib-0042] Moraitou, M. , A. Forsythe , J. A. Fellows Yates , J. C. Brealey , C. Warinner , and K. Guschanski . 2022. “Ecology, Not Host Phylogeny, Shapes the Oral Microbiome in Closely Related Species.” Molecular Biology and Evolution 39: msac263. 10.1093/molbev/msac263. 36472532 PMC9778846

[ece374105-bib-0043] Moraitou, M. , J. Richards , C. Bolyos , et al. 2025. “Host Traits Impact the Outcome of Metagenomic Library Preparation From Dental Calculus Samples Across Diverse Mammals.” *bioRxiv*: 2025.03.19.643754. 10.1101/2025.03.19.643754. PMC12550474 40889349

[ece374105-bib-0044] Naganuma, T. , S. Koike , R. Nakashita , et al. 2020. “Age‐ and Sex‐Associated Differences in the Diet of the Asian Black Bear: Importance of Hard Mast and Sika Deer.” Mammal Study 45, no. 2: 155–166. 10.3106/ms2019-0051.

[ece374105-bib-0045] Nakahama, N. 2021. “Museum Specimens: An Overlooked and Valuable Material for Conservation Genetics.” Ecological Research 36, no. 1: 13–23. 10.1111/1440-1703.12181.

[ece374105-bib-0046] Nolen, Z. J. , P. Jamelska , A. S. T. Lara , N. Wahlberg , and A. Runemark . 2024. “Species‐Specific Loss of Genetic Diversity and Exposure of Deleterious Mutations Following Agricultural Intensification.” *bioRxiv*: 2024–10.

[ece374105-bib-0047] Nourinezhad, J. , A. Moarabi , and M. S. R. Ahkalani . 2021. “Detailed Gross Anatomic and Sialographic Characteristics of Major Salivary Glands in Water Buffaloes ( *Bubalus bubalis* ).” Anatomical Science International 96, no. 3: 427–442. 10.1007/s12565-021-00609-8. 33555557

[ece374105-bib-0048] O'Regan, H. J. , and A. C. Kitchener . 2005. “The Effects of Captivity on the Morphology of Captive, Domesticated and Feral Mammals.” Mammal Review 35, no. 3/4: 215–230. 10.1111/j.1365-2907.2005.00070.x.

[ece374105-bib-0049] Ozga, A. T. , and C. Ottoni . 2023. “Dental Calculus as a Proxy for Animal Microbiomes.” Quaternary International, From Food to Environments: Advances in Ancient Human Dental Calculus Research 653–654: 47–52. 10.1016/j.quaint.2021.06.012. PMC7614904 37559969

[ece374105-bib-0050] Pagel, M. 1999. “Inferring the Historical Patterns of Biological Evolution.” Nature 401: 877–884. 10553904 10.1038/44766

[ece374105-bib-0051] Power, R. C. , D. C. Salazar‐García , L. G. Straus , M. R. González Morales , and A. G. Henry . 2015. “Microremains From El Mirón Cave Human Dental Calculus Suggest a Mixed Plant–Animal Subsistence Economy During the Magdalenian in Northern Iberia.” Journal of Archaeological Science 60: 39–46. 10.1016/j.jas.2015.04.003.

[ece374105-bib-0052] Power, R. C. , D. C. Salazar‐García , R. M. Wittig , M. Freiberg , and A. G. Henry . 2015. “Dental Calculus Evidence of Taï Forest Chimpanzee Plant Consumption and Life History Transitions.” Scientific Reports 5, no. 1: 15161. 10.1038/srep15161. 26481858 PMC4611876

[ece374105-bib-0053] Putrino, A. , E. Marinelli , A. Galeotti , G. F. Ferrazzano , M. Ciribè , and S. Zaami . 2024. “A Journey Into the Evolution of Human Host‐Oral Microbiome Relationship Through Ancient Dental Calculus: A Scoping Review.” Microorganisms 12, no. 5: 902. 10.3390/microorganisms12050902. 38792733 PMC11123932

[ece374105-bib-0054] Qian, H. , and Y. Jin . 2020. “Are Phylogenies Resolved at the Genus Level Appropriate for Studies on Phylogenetic Structure of Species Assemblages?” Plant Diversity 43: 255–263. 10.1016/j.pld.2020.11.005. 34485767 PMC8390917

[ece374105-bib-0055] Radini, A. , E. Nikita , S. Buckley , L. Copeland , and K. Hardy . 2017. “Beyond Food: The Multiple Pathways for Inclusion of Materials Into Ancient Dental Calculus.” American Journal of Physical Anthropology 162, no. S63: 71–83. 10.1002/ajpa.23147. 28105717

[ece374105-bib-0056] Reuter, D. M. , S. S. B. Hopkins , and S. A. Price . 2023. “What Is a Mammalian Omnivore? Insights Into Terrestrial Mammalian Diet Diversity, Body Mass and Evolution.” Proceedings of the Royal Society B: Biological Sciences 290, no. 1992: 20221062. 10.1098/rspb.2022.1062. PMC9890115 36722085

[ece374105-bib-0057] Rivera‐Perez, J. I. , R. J. Cano , Y. Narganes‐Storde , L. Chanlatte‐Baik , and G. A. Toranzos . 2015. “Retroviral DNA Sequences as a Means for Determining Ancient Diets.” PLoS One 10, no. 12: e0144951. 10.1371/journal.pone.0144951. 26660678 PMC4682816

[ece374105-bib-0058] Schindel, D. E. , and J. A. Cook . 2018. “The Next Generation of Natural History Collections.” PLoS Biology 16, no. 7: e2006125. 10.1371/journal.pbio.2006125. 30011273 PMC6062129

[ece374105-bib-0059] Shaiber, A. , A. D. Willis , T. O. Delmont , et al. 2020. “Functional and Genetic Markers of Niche Partitioning Among Enigmatic Members of the Human Oral Microbiome.” Genome Biology 21, no. 1: 292. 10.1186/s13059-020-02195-w. 33323122 PMC7739484

[ece374105-bib-0060] Spellman, G. M. 2019. “The Extended Specimen: Emerging Frontiers in Collections‐Based Ornithological Research.” Auk 136, no. 3: ukz024. 10.1093/auk/ukz024.

[ece374105-bib-0061] Tidière, M. , J.‐M. Gaillard , V. Berger , et al. 2016. “Comparative Analyses of Longevity and Senescence Reveal Variable Survival Benefits of Living in Zoos Across Mammals.” Scientific Reports 6, no. 1: 36361. 10.1038/srep36361. 27819303 PMC5098244

[ece374105-bib-0062] Tucker, R. 1958. “Taxonomy of the Salivary Glands of Vertebrates.” Systematic Zoology 7, no. 2: 74–83. 10.2307/2411794.

[ece374105-bib-0063] Upham, N. S. , J. A. Esselstyn , and W. Jetz . 2019. “Inferring the Mammal Tree: Species‐Level Sets of Phylogenies for Questions in Ecology, Evolution, and Conservation.” PLoS Biology 17, no. 12: e3000494. 10.1371/journal.pbio.3000494. 31800571 PMC6892540

[ece374105-bib-0064] Van Der Valk, T. , D. Díez‐del‐Molino , T. Marques‐Bonet , K. Guschanski , and L. Dalén . 2019. “Historical Genomes Reveal the Genomic Consequences of Recent Population Decline in Eastern Gorillas.” Current Biology 29, no. 1: 165–170.e6. 10.1016/j.cub.2018.11.055. 30595519

[ece374105-bib-0065] Verstraete, F. J. M. 2003. “Advances in Diagnosis and Treatment of Small Exotic Mammal Dental Disease.” Seminars in Avian and Exotic Pet Medicine, Passerine Birds 12, no. 1: 37–48. 10.1053/saep.2003.127877.

[ece374105-bib-0066] Viechtbauer, W. 2010. “Conducting Meta‐Analyses in R With the Metafor Package.” Journal of Statistical Software 36, no. 3: 1–48. 10.18637/jss.v036.i03.

[ece374105-bib-0067] Warinner, C. , J. F. M. Rodrigues , R. Vyas , et al. 2014. “Pathogens and Host Immunity in the Ancient Human Oral Cavity.” Nature Genetics 46: 336–344. 24562188 10.1038/ng.2906 PMC3969750

[ece374105-bib-0068] Warinner, C. , C. Speller , and M. J. Collins . 2015. “A New Era in Palaeomicrobiology: Prospects for Ancient Dental Calculus as a Long‐Term Record of the Human Oral Microbiome.” Philosophical Transactions of the Royal Society, B: Biological Sciences 370, no. 1660: 20130376. 10.1098/rstb.2013.0376. PMC4275884 25487328

[ece374105-bib-0069] Whitehouse‐Tedd, K. M. , S. L. Lefebvre , and G. P. J. Janssens . 2015. “Dietary Factors Associated With Faecal Consistency and Other Indicators of Gastrointestinal Health in the Captive Cheetah ( *Acinonyx jubatus* ).” PLoS One 10, no. 4: e0120903. 10.1371/journal.pone.0120903. 25830636 PMC4382097

[ece374105-bib-0070] Winker, K. 2004. “Natural History Museums in a Postbiodiversity Era.” Bioscience 54, no. 5: 455–459. 10.1641/0006-3568(2004)054[0455:NHMIAP]2.0.CO;2.

[ece374105-bib-0071] Yaprak, E. , İ. Yolcubal , A. Sinanoğlu , A. Doğrul‐Demiray , E. Guzeldemir‐Akcakanat , and İ. Marakoğlu . 2017. “High Levels of Heavy Metal Accumulation in Dental Calculus of Smokers: A Pilot Inductively Coupled Plasma Mass Spectrometry Study.” Journal of Periodontal Research 52, no. 1: 83–88. 10.1111/jre.12371. 27016267

[ece374105-bib-0072] Yeates, D. K. , A. Zwick , and A. S. Mikheyev . 2016. “Museums Are Biobanks: Unlocking the Genetic Potential of the Three Billion Specimens in the World's Biological Collections.” Current Opinion in Insect Science, Neuroscience * Special Section on Insect Phylogenetics 18: 83–88. 10.1016/j.cois.2016.09.009. 27939715

