# Supplementary material for: Dental Calculus Formation Is Linked to Diet and Phylogeny in Mammals
Source: Ecol Evol. 2026 Jul 24;16(7):e74105. doi: 10.1002/ece3.74105 (PMC13400996; doi:10.1002/ece3.74105)
Supplement: Supplementary file 1 — Figure S1: ece374105‐sup‐0001‐FiguresS1‐S8.docx. [file ECE3-16-e74105-s002.docx]

**Supplementary Figures**

**
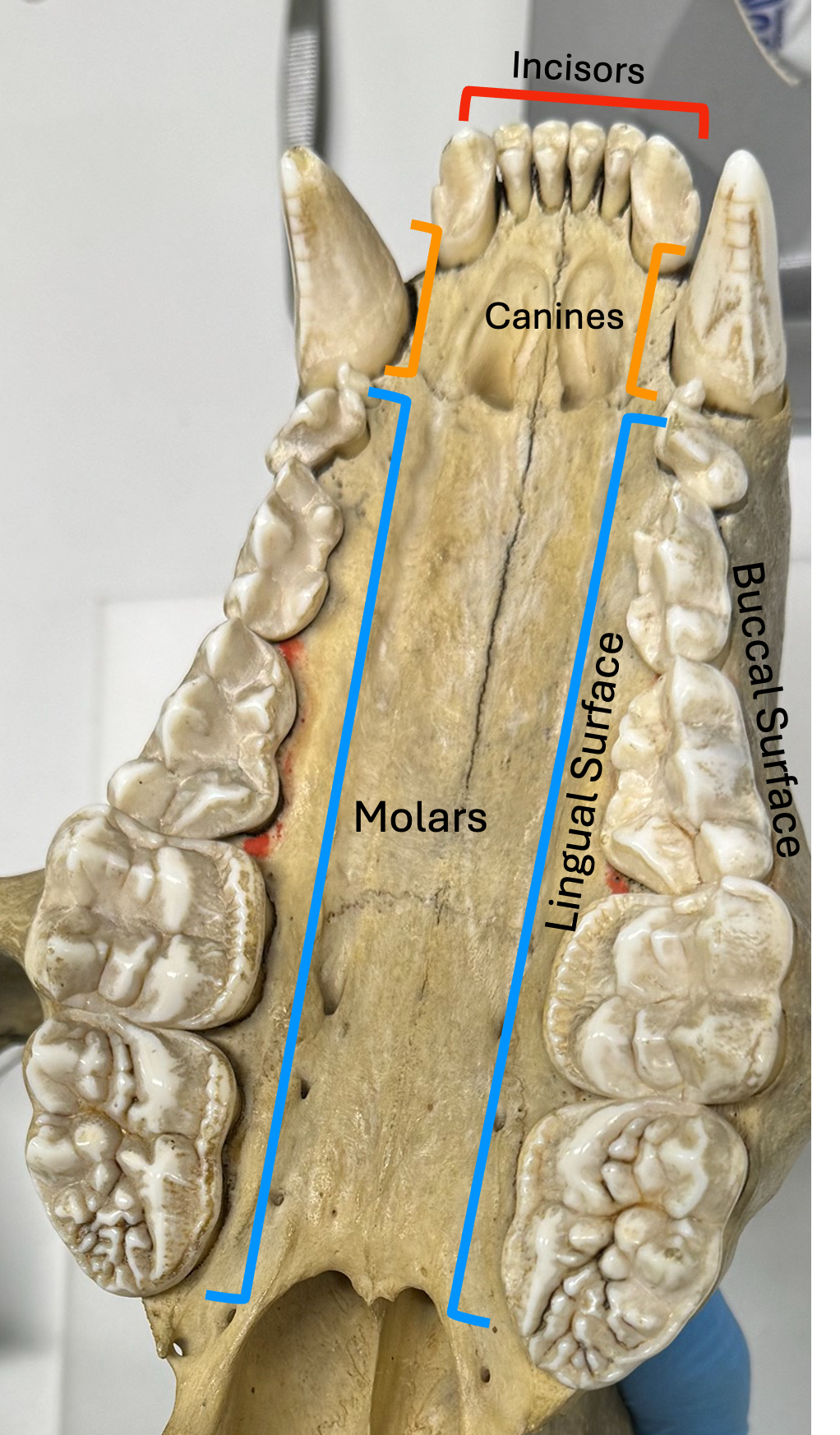
**

**Figure S1: Schematic of tooth side and type.** Exemplary upper jaw of *Ailuropoda melanoleuca* (giant panda). Teeth were scored as incisors (red), canines (orange), and molars (blue, containing both premolars and molars). Tooth surface classified as buccal (cheek-facing) and lingual (tongue-facing). Occlusal face (biting/chewing surface) was not scored.

**
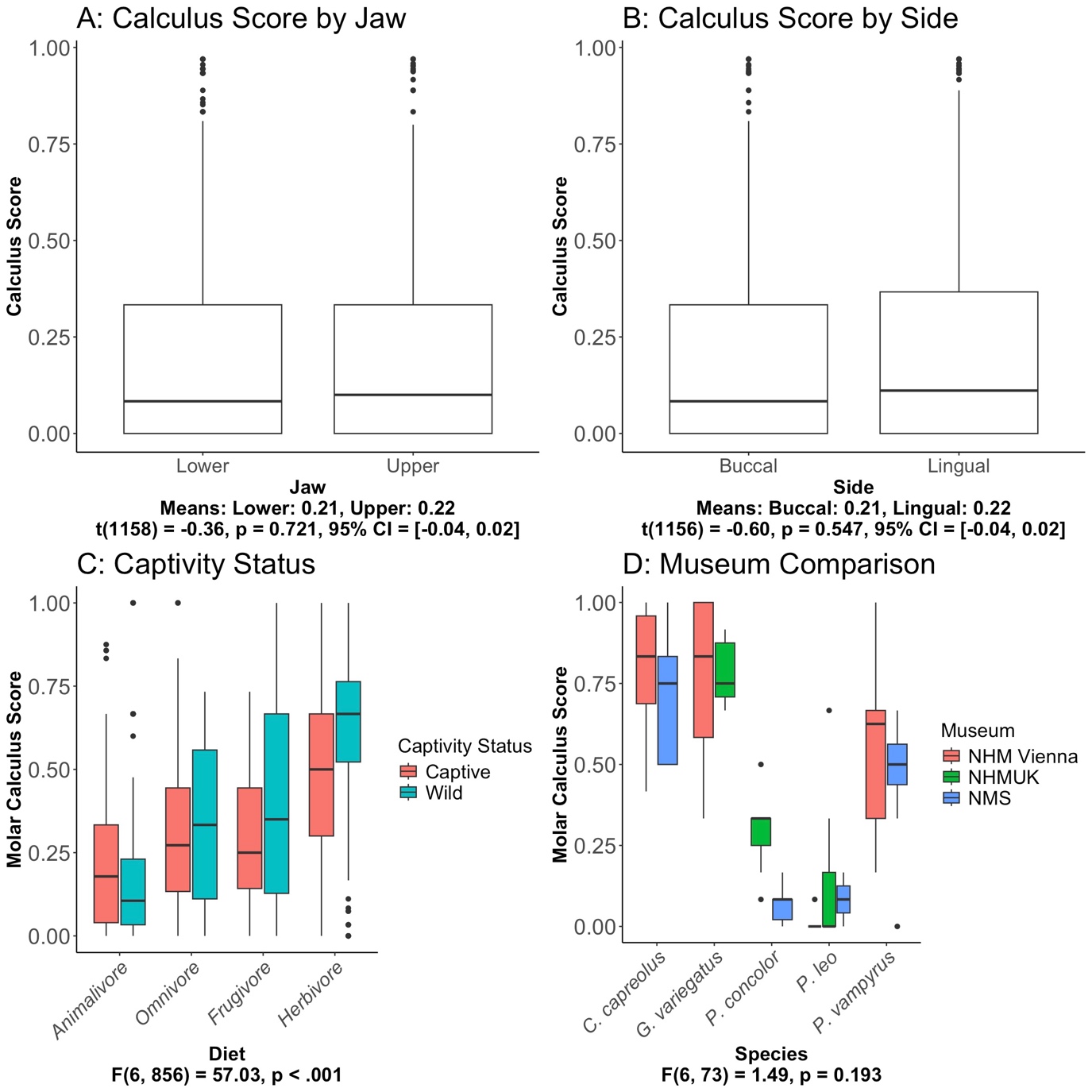
Figure S2: Comparison of calculus scores by jaw (A), side (B), captivity status (C), and museum (D).** Y axes show values for DC scores, considering only molars in C and D. Results for ANOVAs (one-way: A & B, nested: C & D) shown below x axis labels. In D, considered species are *Capreolus capreolus* (roe deer, herbivore), *Galeopterus variegatus* (Sunda colugo, herbivore), *Panthera leo* (lion, animalivore), *Pteropus vampyrus* (large flying fox, frugivore) and *Puma concolor* (puma, animalivore). Values for post hoc tests (including p-values corrected for multiple comparisons) can be found in Table S3.


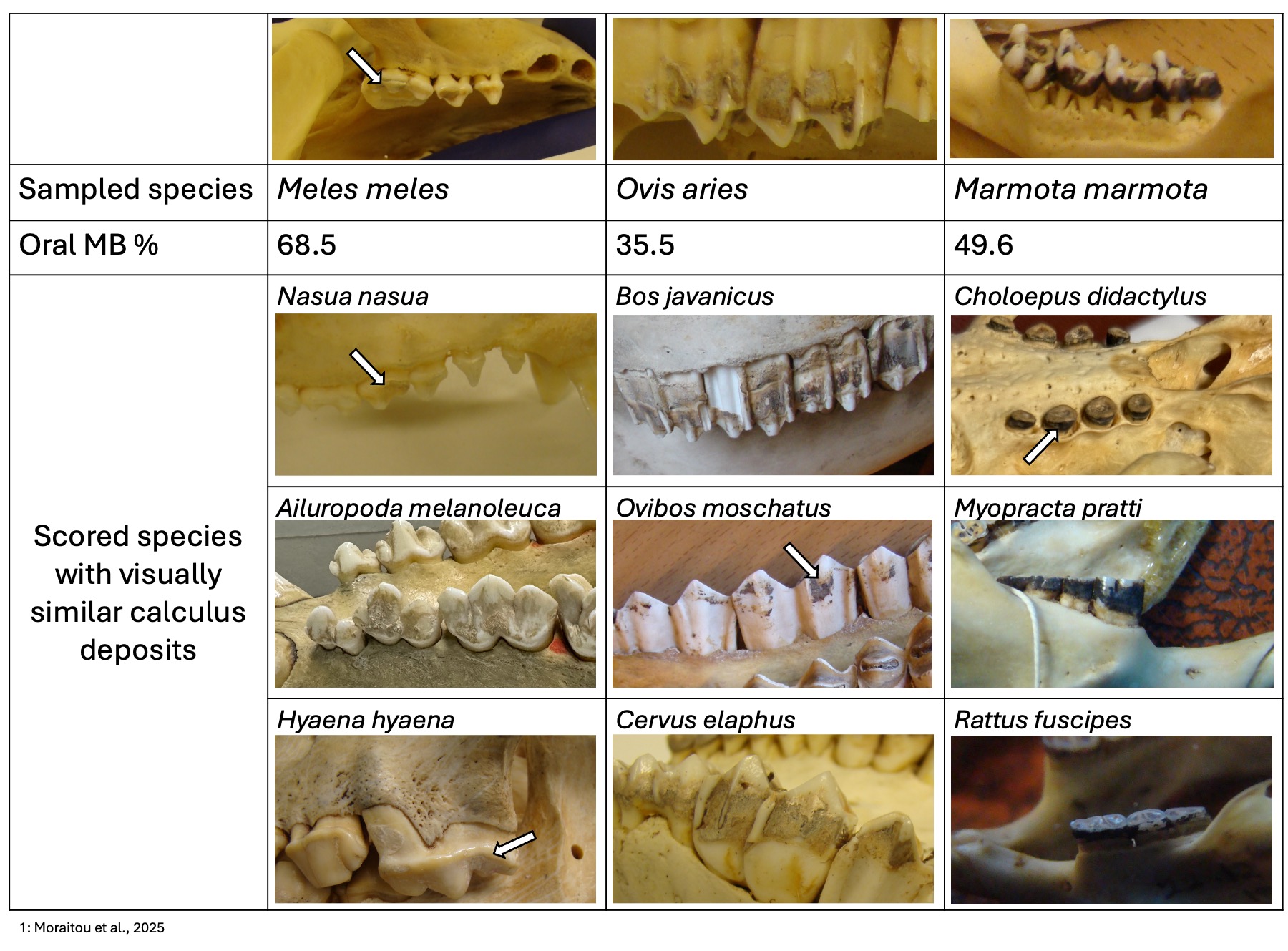


**Figure S3: Morphological diversity of calculus deposits**. Left column: white or pale, chalky plaques frequently observed on omnivores and carnivores; Central column: darker, chalky, layered plaques, sometimes present on herbivores and particularly ruminants; Right column: dark film, frequently covering the entire supragingival portion of the tooth, often found on herbivores. Top row shows images of calculus from species in which the composition of the deposit was confirmed as oral microbiome using metagenomic sequencing (Moraitou, et al., 2025), also reporting the determined oral microbiome proportion (Oral MB %) that resembles human oral microbiome of the analyzed metacommunity.


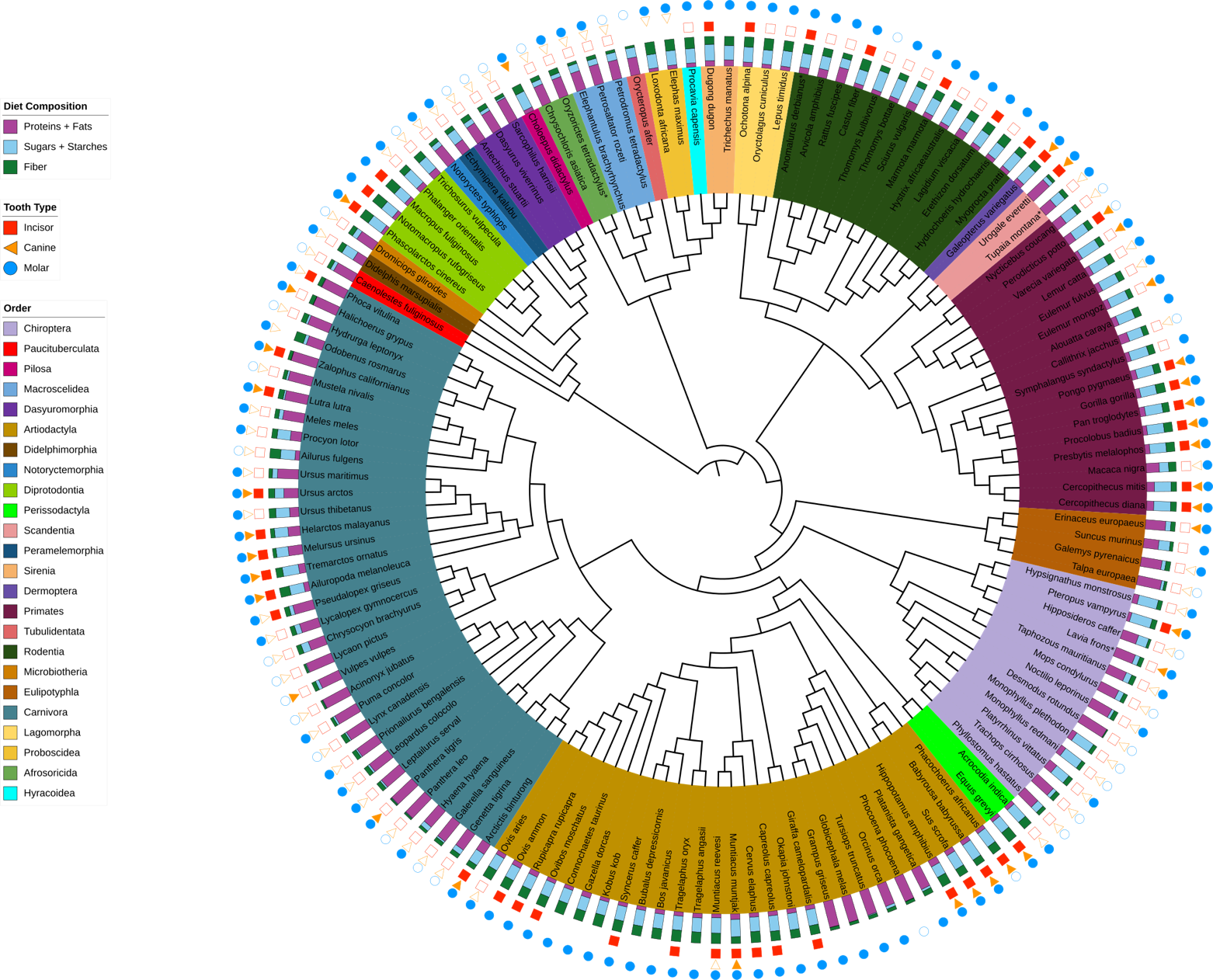


**Figure S4: Dental calculus presence across the mammalian phylogeny and tooth types, showing all surveyed wild species (n=141).** Mean scores per tooth type were binarized, with a score >0.1 considered as DC presence and shown with a filled shapes along the three outer rings. Unfilled shapes indicate a mean score ≤0.1, considered as absence of DC. No shape indicates that the tooth type was absent for this species. All odontoceti teeth were scored as molars. This tree was downloaded from vertlife.org (Upham et al., 2019) using the MCC consensus tree of DNA-only records. Asterisks indicate surveyed species not present in the downloaded tree. For *Anomalurus derbianus, Tupaia montana,* and *Oryzorictes tetradactylus* we used congeneric species as representatives: *A. beecrofti, T. glis, O. hova*.

**Figure S5: Differences in calculus scores accompanying diet changes in close relatives.** Examples taken from the binarized survey phylogeny in Fig. 2. Note the changes in diet (specifically reduced protein intake [purple] and increased fibers, sugars, and starches) relative to their closest surveyed relatives in spiny bandicoot *Echymipera kalubu* (A), binturong *Arctictis binturong* (B), and brown bear *Ursus arctos* (C) and accompanying increase in DC formation.


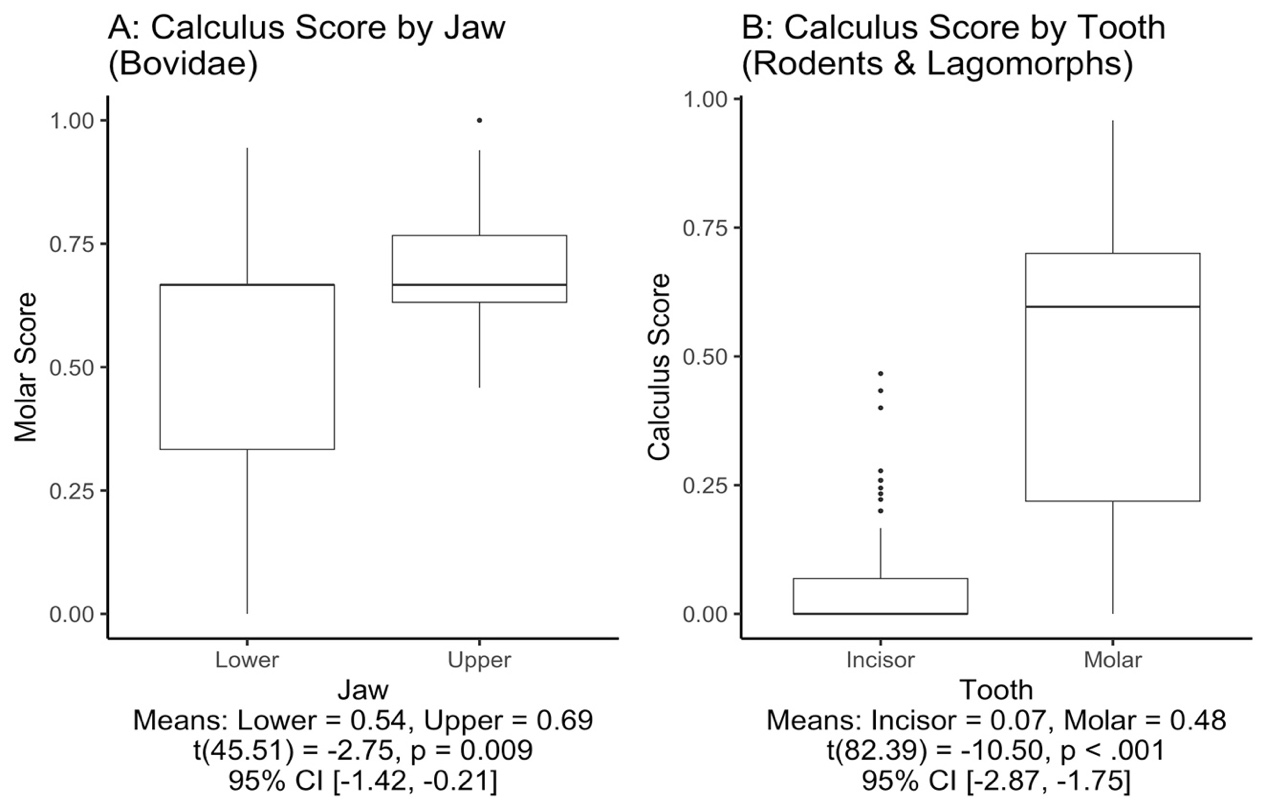


**Figure S6: Comparison of calculus scores in bovids and rodents and lagomorphs.** Results from Welch’s t-test shown below x axis.


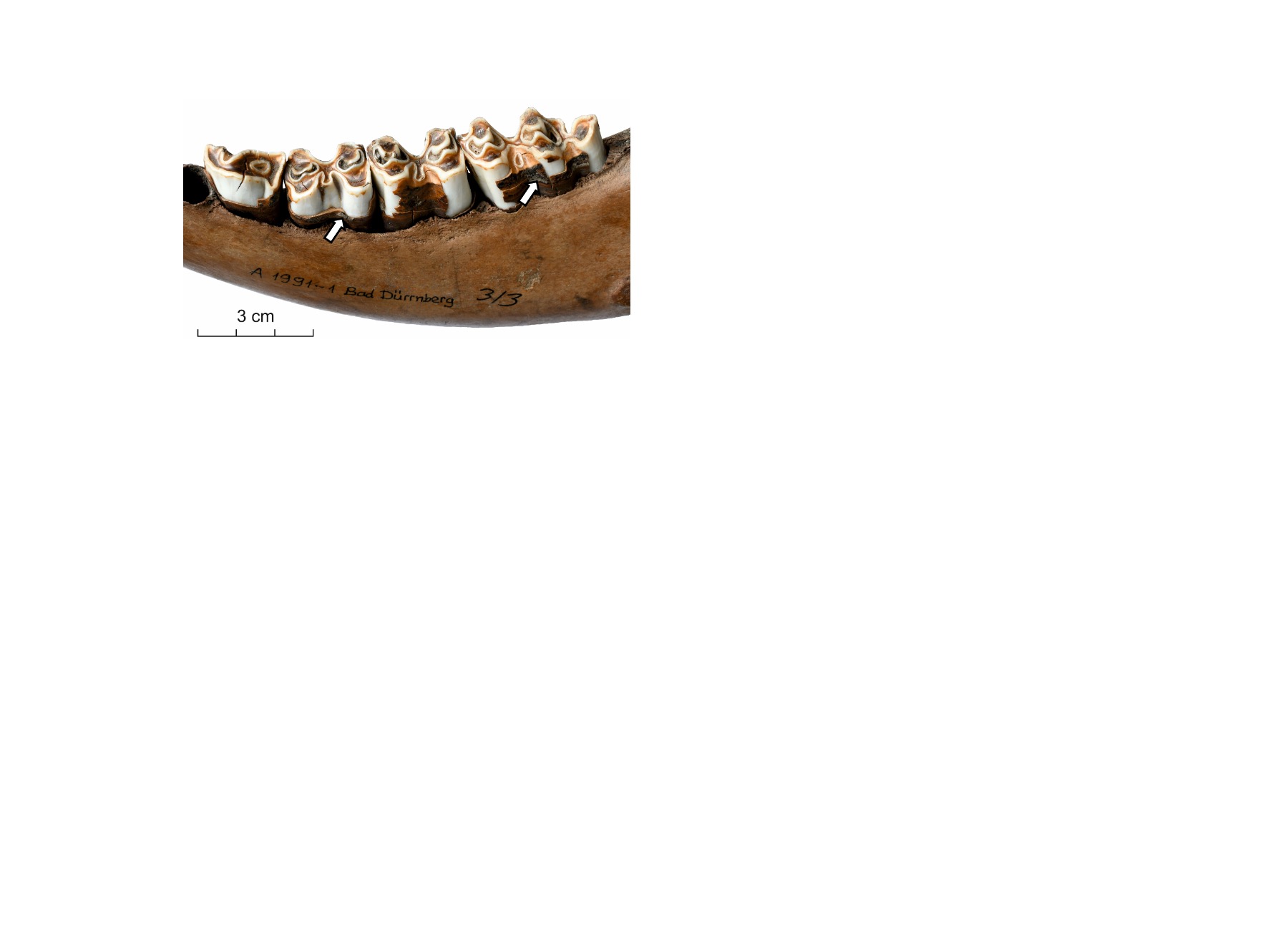


**Figure S7: Dental calculus found on Iron Age cattle jaw.** Specimen recovered from Bad Dürrnberg, Austria. Calculus deposits indicated with white arrows. Note that the smooth brown layer on the teeth corresponds to enamel and not calculus, with calculus showing rough, whitish surface. Specimen is dated to the Early La Tène to Late La Tène period (c. 450 B.C. to 1 B.C.). Specimen taken from the Archeozoological collection of NHM Vienna (Inventory number: A 1991-1).


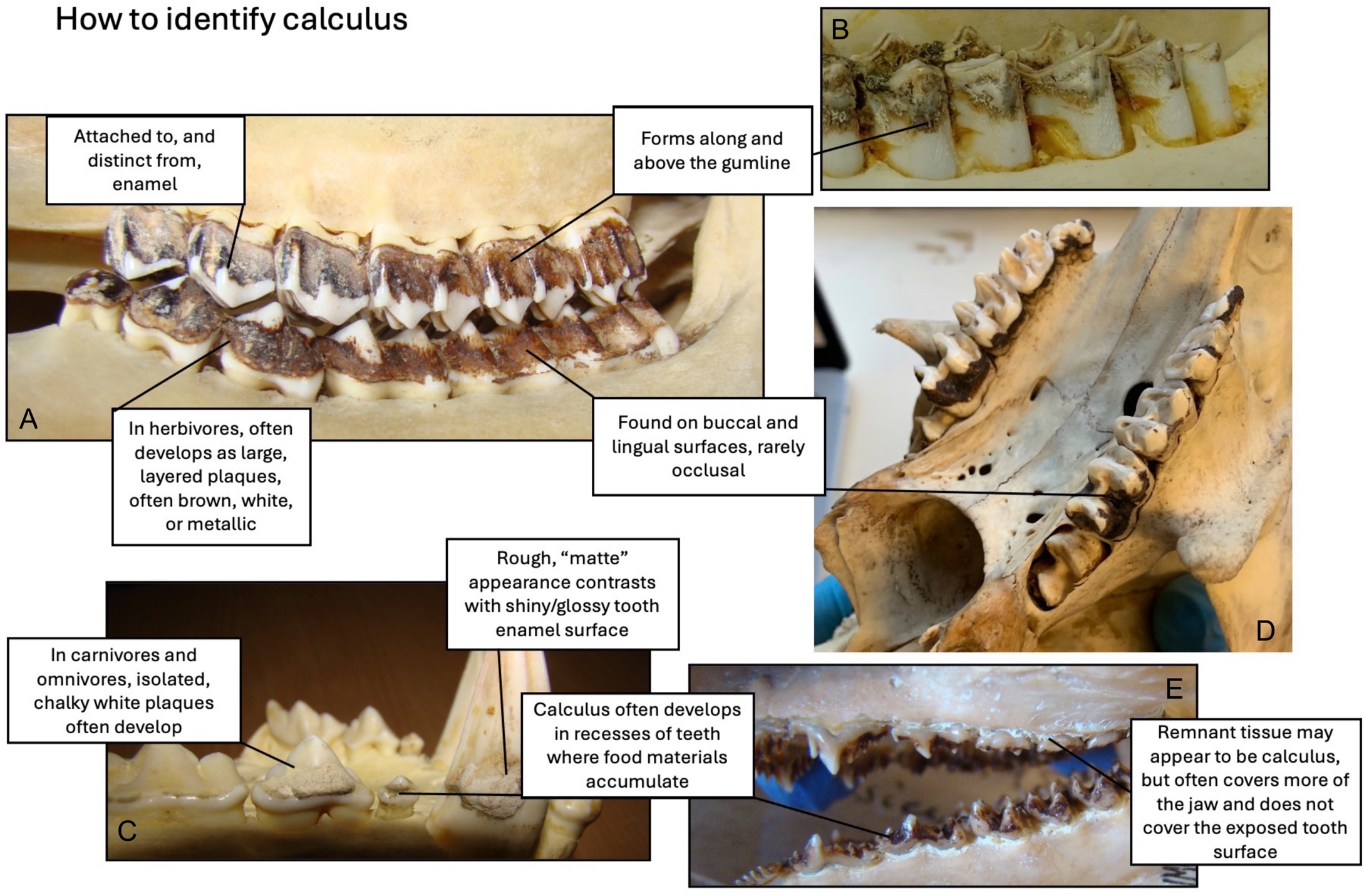


**Figure S8: Further photos of calculus from a range of species with annotations indicating common diagnostic criteria and suggestions for calculus identification across a range of calculus morphologies, species, and diet groups.** A: *Capreolus capreolus,* herbivore; B: *Cervus elaphus,* herbivore; C: *Panthera tigris,* carnivore; D: *Macropus fulginosus,* herbivore; E: *Galeopterus variegatus,* frugivore.
